# Nucleolar-nucleoplasmic shuttling of TARG1 and its control by DNA damage-induced poly-ADP-ribosylation and by nucleolar transcription

**DOI:** 10.1038/s41598-018-25137-w

**Published:** 2018-04-30

**Authors:** Mareike Bütepage, Christian Preisinger, Alexander von Kriegsheim, Anja Scheufen, Eva Lausberg, Jinyu Li, Ferdinand Kappes, Regina Feederle, Sabrina Ernst, Laura Eckei, Sarah Krieg, Gerhard Müller-Newen, Giulia Rossetti, Karla L. H. Feijs, Patricia Verheugd, Bernhard Lüscher

**Affiliations:** 1 Institute of Biochemistry and Molecular Biology, Medical School, RWTH Aachen University, Pauwelsstraße 30, 52074 Aachen, Germany; 2 Proteomics Facility, Interdisciplinary Centre for Clinical Research (IZKF), Medical School, RWTH Aachen University, Pauwelsstraße 30, 52074 Aachen, Germany; 3 Systems Biology Ireland, Conway Institute, University College Dublin, Dublin 4, Ireland; 4 Institute for Diabetes and Obesity, Monoclonal Antibody Core Facility, Helmholtz Center Munich, German Research Center for Environmental Health, Ingolstädter Landstrasse 1, Neuherberg, Germany; 5 Immunohistochemistry and Confocal Microscopy Facility, Interdisciplinary Centre for Clinical Research (IZKF), Medical School, RWTH Aachen University, Pauwelsstraße 30, 52074 Aachen, Germany; 6 Computational Biomedicine, Institute for Advanced Simulation IAS-5 and Institute of Neuroscience and Medicine INM-9, Forschungszentrum Jülich, 52425 Jülich, Germany; 7 Jülich Supercomputing Centre, Forschungszentrum Jülich, 52425 Jülich, Germany; 8 Department of Oncology, Hematology and Stem Cell Transplantation, Medical School, RWTH Aachen University, Pauwelsstraße 30, 52074 Aachen, Germany; 9 Present Address: Edinburgh Cancer Research Centre, IGMM, University of Edinburgh, Edinburgh, EH4 2XR UK; 10 Present Address: Institute of Human Genetics, Medical School, RWTH Aachen University, Pauwelsstraße 30, 52074 Aachen, Germany; 11 Present Address: College of Chemistry, Fuzhou University, 350116 Fuzhou, China; 12 Present Address: Department of Biological Sciences, Xi’an Jiaotong-Liverpool University, No 111, Ren Ai Road, Dushu Lake Higher Education Town, Suzhou Industrial Park, Suzhou, 215123 P.R. China

## Abstract

Macrodomains are conserved protein folds associated with ADP-ribose binding and turnover. ADP-ribosylation is a posttranslational modification catalyzed primarily by ARTD (aka PARP) enzymes in cells. ARTDs transfer either single or multiple ADP-ribose units to substrates, resulting in mono- or poly-ADP-ribosylation. TARG1/C6orf130 is a macrodomain protein that hydrolyzes mono-ADP-ribosylation and interacts with poly-ADP-ribose chains. Interactome analyses revealed that TARG1 binds strongly to ribosomes and proteins associated with rRNA processing and ribosomal assembly factors. TARG1 localized to transcriptionally active nucleoli, which occurred independently of ADP-ribose binding. TARG1 shuttled continuously between nucleoli and nucleoplasm. In response to DNA damage, which activates ARTD1/2 (PARP1/2) and promotes synthesis of poly-ADP-ribose chains, TARG1 re-localized to the nucleoplasm. This was dependent on the ability of TARG1 to bind to poly-ADP-ribose. These findings are consistent with the observed ability of TARG1 to competitively interact with RNA and PAR chains. We propose a nucleolar role of TARG1 in ribosome assembly or quality control that is stalled when TARG1 is re-located to sites of DNA damage.

## Introduction

ADP-ribosylation is a reversible post-translational modification and involves the transfer of ADP-ribose (ADPr) units from the cofactor NAD^+^ onto substrate proteins. In cells, ADP-ribosylation is catalyzed by the ADP-ribosyltransferase (ART) family, referred to as ART diphtheria toxin-like or ARTD enzymes (aka PARPs)^1,2^. Mono-ADP-ribosylation (MARylation), the transfer of a single ADPr unit to substrates, is catalyzed by the majority of ARTD enzymes and regulates a variety of cellular processes such as cell proliferation, signaling and transcription^3^. In poly-ADP-ribosylation (PARylation) reactions, multiple ADPr moieties are transferred to a substrate in an iterative manner, resulting in modification by long, sometimes branched ADPr chains. PARylation is catalyzed by ARTD1, 2, 5 and 6 (PARP1 and 2, Tankyrase 1 and 2, respectively). ARTD1/2-mediated PARylation plays important roles in cellular stress signaling pathways and auto-modification of ARTD1/2 and PARylation of histones and other chromatin-associated proteins occurs quickly in response to DNA damage^2,4^. Moreover, PAR chains provide binding sites for DNA repair and chromatin remodeling factors, promoting efficient repair^2^. These interactions are mediated by a number of PAR binding domains, including macrodomains. Protein PARylation after DNA damage is of transient nature and PAR chains are quickly degraded by PARG (poly-ADP-ribose glycohydrolase), the catalytic function of which is mediated by a macrodomain^5^.

Macrodomains are structurally conserved protein domains of 130–190 amino acids found in eukaryotes, prokaryotes and viruses^6,7^. Macrodomains adopt a globular α/β/α-sandwich fold and possess a pocket for binding to ADPr or other NAD^+^-derived metabolites such as *O*-acetyl-ADPr (OA-ADPr). Macrodomains differ in their affinities, substrate specificities and activities, interacting specifically with either free and/or protein-bound ADPr or with PAR chains, or both. Some macrodomains exert hydrolytic activity towards ADP-ribosylated proteins. Macrodomains are therefore central to ADPr biology serving as either binding modules for ADP-ribosylated proteins or as hydrolases of ADP-ribosylation. PARG degrades PAR chains by hydrolyzing the glycosidic bond between two ribose sugars but does not cleave the linkage between the protein-proximal ADPr residue and the modified amino acid^5^. Thus, to completely reverse ADP-ribosylation, specific mono-ADPr-hydrolases are required. Three of the 11 human macrodomain-containing proteins known thus far, MacroD1, MacroD2 and TARG1/C6orf130, have been characterized as mono-ADPr-specific hydrolases, reverting glutamate/aspartate-linked MARylation^6,8–11^. While MacroD1 and MacroD2 are closely related at the sequence level (MacroD-type macrodomains), TARG1 is unique among the macrodomain-containing MAR hydrolases, as it is phylogenetically related to ALC-type macrodomains^7^. TARG1 is postulated to employ a different catalytic mechanism compared to the MacroD-type hydrolases^9^. In addition to hydrolysis of MARylation, TARG1 exerts OA-ADPr deacetylase activity^12^ and can remove whole PAR chains from PARylated ARTD1^9^, the latter representing a unique, but rather weak activity among all so far identified MAR hydrolases^9,13^. In cells, TARG1 interacts with ARTD1 and is recruited to sites of laser-induced DNA damage dependent on PARylation^9^. TARG1 depletion from HEK293 and U2OS cells was suggested to increase senescence and sensitivity to DNA damaging agents, indicating a role in DNA repair^9^. An inactivating homozygous mutation in the TARG1-encoding *OARD1* gene has been correlated with childhood neurodegeneration^9^.

Although now generally accepted as an ADPr binding module, macrodomains possess a variety of binding properties beyond ADPr or its directly related metabolites. At least some macrodomains interact with long negatively charged polymers, which can be PAR but also poly(A)^+^ RNA, other single stranded (ss) RNA molecules, or oligo(G) nucleotides^14–18^. Binding of these polymers including PAR is not necessarily mediated by interaction with the ADPr binding pocket, but rather appears to involve interaction with positively charged patches on the surface of the macrodomains^14^.

While addressing the role of TARG1 in regulating chromatin, we noticed that the protein is predominantly located in nucleoli. Therefore, we characterized the TARG1 interactome. Ribosomal proteins and proteins associated with rRNA metabolism and RNA binding were the main interaction partners. However, when ARTD1/2 were activated in cell extracts, a strong shift in the interactome towards PARylated proteins was noticed. Furthermore, we observed that TARG1 shuttles continuously between nucleoli and the nucleoplasm and accumulates in transcriptionally active nucleoli under steady-state conditions. Upon DNA damage rapid and reversible relocation into the nucleoplasm occurred, which was dependent on the ADPr binding ability of TARG1. The accumulation in nucleoli and PARylation-dependent relocation to the nucleoplasm are consistent with the ability of TARG1 to bind RNA and PAR in a competitive manner. In conclusion, we propose that TARG1 is a nucleolar ribosome biosynthesis quality control factor.

## Results

### Tandem-affinity purification reveals interaction of TARG1 with RNA-binding proteins

**Figure 1:**
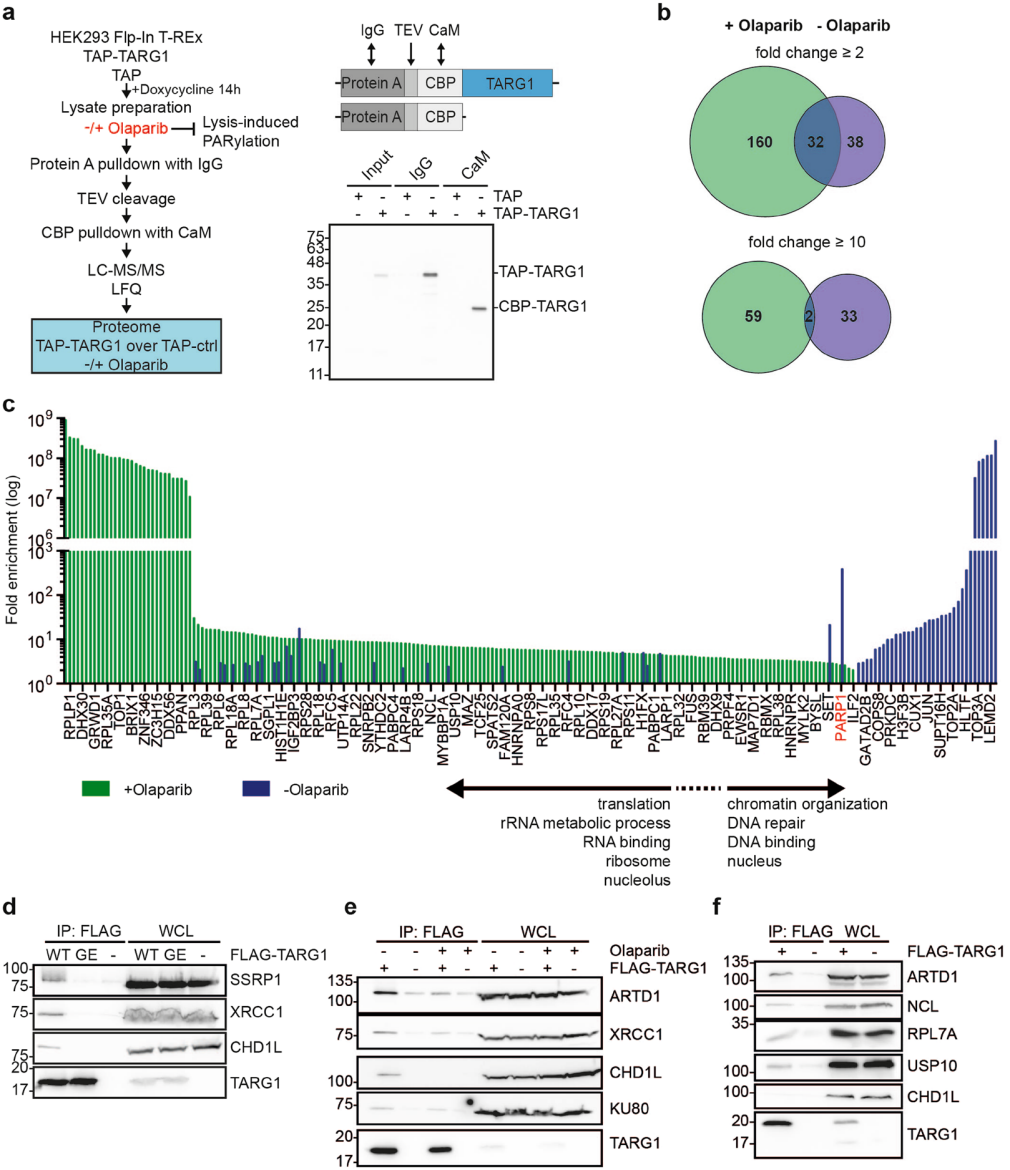
Tandem affinity purification of TARG1-containing protein complexes suggests functions of TARG1 in RNA biology. (**a**) Left panel: HEK293 Flp-In™ T-REx™ cells expressing TAP-TARG1 or the TAP-tag alone after doxycycline induction were lysed in the presence or absence of olaparib to inhibit ARTD1/2 activation during cell lysis. TAP-tag containing complexes were purified in two sequential affinity purification steps. Co-purified proteins were analyzed by LC-MS/MS and relative enrichment of TAP-TARG1 over TAP-tag control was determined by label-free quantitation (LFQ). Upper right panel: Domain structure of TAP-TARG1 and TAP constructs. Lower right panel: Fractions of the input lysate, the IgG and the CaM pulldown were analyzed for TARG1 by Western blot using mAb 3A5 (for characterization of TARG1 antibodies see Supplementary Fig. S3). (**b**) Venn diagrams depicting the overlap between proteins associated with TARG1 in proteomics screens conducted in the absence (three biological replicates, p ≤ 0.01) or presence (two biological replicates, p ≤ 0.01) of olaparib in the lysis buffer with a fold enrichment of ≥2 (upper diagram) or ≥10 (lower diagram) over TAP control. (**c**) Fold enrichment of all proteins identified as TARG1 interacting proteins (p ≤ 0.01, ≥2-fold enrichment over TAP control) both in the absence and presence of olaparib. Only a fraction of grouped bars is specified with the corresponding protein names on the x-axis for clarity reasons. For details on the identified proteins see Supplementary Table S1, Supplementary Datasets S1 and S2. (**d**) HEK293 cells were transfected with plasmids encoding for FLAG-TARG1 wildtype (WT) or ADPr binding-deficient FLAG-TARG1-G123E (GE). Presence of SSRP1, XRCC1 and CHD1L in FLAG immunoprecipitates (IP) and whole cell lysates (WCL) was analyzed by Western blotting. (**e**) HEK293 cells transiently expressing FLAG-TARG1 were lysed in TAP lysis buffer ±10 µM olaparib. Presence of ARTD1, XRCC1, CHD1L, KU80 and TARG1 after IP of FLAG-TARG1 was analyzed by Western blotting. (**f**) HEK293T cells transiently expressing FLAG-TARG1 were lysed in the presence of 10 µM olaparib. Presence of ARTD1, NCL, RPL7A and USP10 after IP against FLAG-TARG1 was analyzed by Western blotting. CHD1L was detected to control for inhibition of lysis-induced PARylation. (**d–f**) Full-length blots are presented in Supplementary Fig. S8.

To gain insight into TARG1’s cellular functions, we identified the TARG1-associated cellular proteome using a tandem affinity purification (TAP) approach^19^. HEK293 cells stably and inducibly expressing TAP-tagged TARG1 or the TAP-tag alone were generated and TAP-containing protein complexes isolated (Fig. 1a)^20^. The TAP-tag consists of Protein A fused to a Calmodulin (CaM) binding peptide (CBP) via a Tobacco Etch Virus (TEV) protease cleavage site (Fig. 1a), allowing for sequential affinity purification of TAP-tag-containing complexes. Protein A is captured by an IgG matrix, complexes are eluted by TEV cleavage and CBP-tagged complexes are recovered by a CaM pulldown (Fig. 1a). Co-purified proteins were analyzed by LC-MS/MS and relative enrichment of detected proteins in the TAP-TARG1 pulldown over the TAP-tag control was calculated by label-free quantitation (Fig. 1b and c)^21,22^. Because mechanical DNA shearing during cell lysis activates ARTD1/2 resulting in PAR formation, to which TARG1 can be recruited^9,23^, we assessed the role of PAR on the TARG1 interactome. Therefore, the experiments were performed with or without the ARTD1/2 inhibitor olaparib during cell lysis (Fig. 1a). Experiments without inhibitor were performed in three, experiments with inhibitor in two biological replicates. The individual samples were measured in technical duplicates. Without inhibitor we identified 70 TARG1 interacting proteins with a fold enrichment ≥2 (Fig. 1b, Supplementary Table S1, Supplementary Dataset S1). ARTD1, the only described interaction partner of TARG1 so far^9^, was highly enriched (Fig. 1c; Supplementary Table S1, Supplementary Dataset S1). In addition, components of protein complexes involved in base excision repair (BER; XRCC1 and LIG3), in non-homologous end joining (NHEJ; PRKDC, XRCC5 and XRCC6) and the FACT (facilitates chromatin transcription) complex subunits SSRP1 and SUPT16H were associated with TARG1. All of these factors have been consistently found as proteins that are PARylated in response to DNA damage or are recruited to sites of DNA damage via PAR binding domains^23,24^.

To assess whether these interactions depend on PARylation, we generated different ADPr binding-deficient TARG1 mutants. Based on *in silico* mutational analyses of the ADPr binding pocket in TARG1, we mutated three residues located in the ADPr binding pocket, G43, I44 and G123, to glutamate (Supplementary Fig. S1a–c, Supplementary Table S2). Mutation of G123 was previously shown to block ADPr binding^9^. Impact of the mutations on folding and ADPr binding was assessed in fluorescence-based thermal shift assays^25^. His-TARG1-G43E and -I44E were less, His-TARG1-G123E more stable than the wildtype protein (Supplementary Fig. S1d). While His-TARG1 was stabilized by ADPr, the mutants were not, indicative of loss of ADPr binding (Supplementary Fig. S1d). Moreover, all three mutants were catalytically inactive towards auto-modified ARTD10 (Supplementary Fig. S1e). Consistent with these findings and unlike the wildtype protein, TARG1-G123E did not interact with repair proteins in co-immunoprecipitation (co-IP) experiments (Fig. 1d).

In the presence of olaparib a strong shift was observed in TARG1 co-purified proteins (Fig. 1b). The +olaparib dataset contained 192 proteins with a ≥2-fold enrichment with an overlap of 32 proteins to the –olaparib dataset (Fig. 1b). The lost proteins with olaparib included those being either substrates of or recruited by DNA damage-induced PARylation (Fig. 1c and e). Increasing the stringency to a threshold of ≥10 resulted in an overlap of only one protein, RFC2 (replication factor C subunit 2), in addition to TARG1 itself (Fig. 1b). In the presence of olaparib, we identified almost all proteins of the small and large ribosomal subunits and proteins involved in ribosome biogenesis, RNA processing and translation such as nucleolin (NCL), hnRNPs and DDX proteins (Fig. 1c, Supplementary Table S1, Supplementary Dataset S2). Among the ARTD family members, association of TARG1 with ARTD1 and the catalytically inactive ARTD13 was observed. However, while ARTD1 was highly enriched in the absence of olaparib treatment (385-fold), it was only weakly interacting (2.6-fold) with TARG1 with ARTD1/2 inhibition (Fig. 1c, Supplementary Table S1, Supplementary Dataset S2). Independent co-IP experiments using FLAG-tagged TARG1 from cell lysates in the presence of olaparib confirmed binding to ARTD1, NCL, RPL7A, and USP10 (Fig. 1f). CHD1L was not co-IPed, indicating that lysis-induced PAR formation was very low or did not occur in these experiments (Fig. 1f). We were not able to IP endogenous TARG1 under co-IP conditions with any of our monoclonal and polyclonal antibodies despite their reactivity with endogenous TARG1 on Western blots and in high stringency IP buffers (for control Western blots see below). A possible explanation is that the epitopes of this small protein are covered by interacting factors under low stringency conditions.

In summary, we observed a shift of proteins co-purifying with TARG1, depending on the addition of the PARP inhibitor olaparib to the lysis buffer (Fig. 1b and c). Gene ontology (GO) analyses revealed distinct biological and molecular functions associated with these proteins (Fig. 1c, Supplementary Table S3). Under –olaparib conditions, chromatin-related processes such as DNA repair, chromatin organization and DNA binding were overrepresented, while with inhibition of ARTD1/2 RNA-related processes such as translation, rRNA metabolic process and RNA binding prevailed (Fig. 1c, Supplementary Table S3).

### TARG1 does not affect cell proliferation or translation of HeLa cells

**Figure 2:**
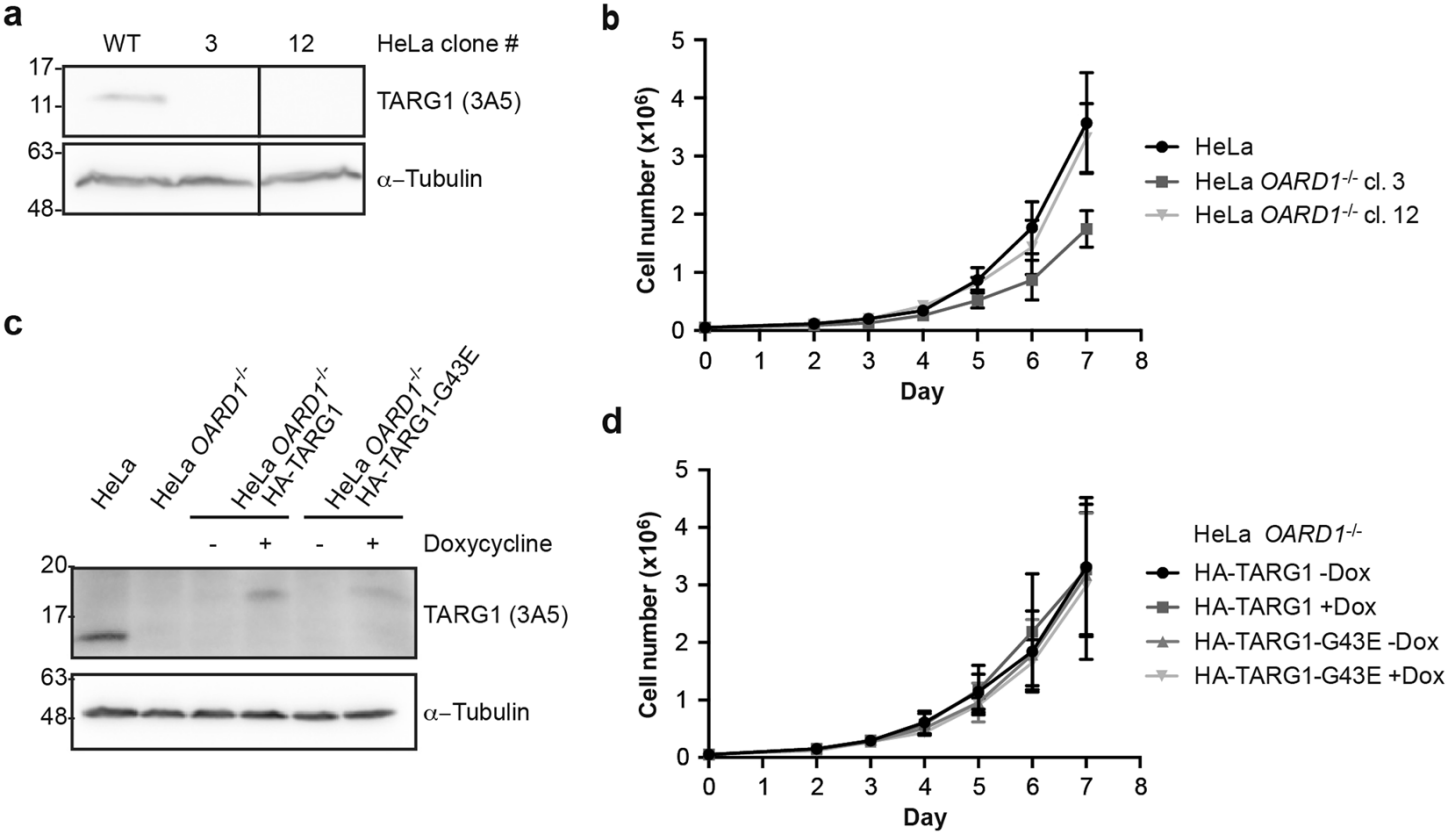
*OARD1* knock-out does not affect cell proliferation of HeLa cells. (**a**) TARG1 expression was analyzed in whole cell lysates of HeLa cells (WT) and the HeLa *OARD1*^−/−^ clones 3 and 12 by Western blotting using the TARG1-specific monoclonal antibody 3A5. α-Tubulin was detected as loading control. (**b**) Cell proliferation of HeLa cells and HeLa *OARD1*^−/−^ clones 3 and 12 was measured by counting living cells over 7 days. (**c**) HeLa *OARD1*^−/−^ (cl. 3) cells were stably transduced using a lentiviral vector allowing for doxycycline-inducible re-expression of HA-TARG1 or HA-TARG1-G43E. TARG1 protein expression was analyzed in HeLa *OARD1*^−/−^ HA-TARG1 wildtype or -G43E cells that were treated with 100 ng/ml doxycycline or were left untreated for 48 h by Western blotting. α-Tubulin levels were detected as a loading control. (**d**) Cell proliferation of HeLa *OARD1*^−/−^ HA-TARG1 or HA-TARG1-G43E cells, which were treated with 100 ng/ml doxycycline every 48–72 h or were left untreated (±Dox), was measured over 7 days by counting living cells. (**a** and **c**) Full-length blots are presented in Supplementary Fig. S8.

To evaluate a potential role of TARG1 in cell proliferation and translation, we established HeLa *OARD1*^−/−^ cells using the CRISPR-nCas9 system^26^. We designed four gRNAs targeting nCas9 to loci in introns 2 and 5 of the *OARD1* gene, resulting in the deletion of a fragment comprising exons 3–5 (Supplementary Fig. S2a). We identified two clones with the desired deletion in the *OARD1* gene by genomic PCR (Supplementary Fig. S2b). No residual TARG1 protein was detected in lysates of the HeLa knock-out clones by Western blot analyses using both mAbs and pAbs (Fig. 2a; antibodies are described in Supplementary Fig. S3).

We measured cell proliferation of the two HeLa *OARD1*^−/−^ clones by counting living cells over 7 days. While clone 12 HeLa *OARD1*^−/−^ cells proliferated as fast as wildtype cells, the proliferation rate of cells generated from clone 3 was reduced by half (Fig. 2b). The reduction of cell proliferation observed for HeLa *OARD1*^−/−^ clone 3 was not caused by the loss of TARG1, as complementation with a lentiviral vector expressing HA-TARG1 wildtype or HA-TARG1-G43E did not affect proliferation (Fig. 2c and d). Similarly, substantial overexpression of TARG1 or TARG1-G123E or shRNA-dependent knockdown of *OARD1* in HeLa Flp-In™ T-REx™ cells did neither affect cell proliferation nor sensitivity to DNA damage-inducing agents (Supplementary Figs S4 and S5). In summary, although it had been observed previously that TARG1 has growth promoting functions in HEK293/293T, NIH3T3, HSC58 and HSC60 cells^9,27^, our findings suggest that TARG1 does not affect cell proliferation in HeLa cells.

Because of the strong enrichment of ribosomal proteins in the TARG1-associated proteome, we next addressed whether TARG1 plays a role in translation. Cells lacking TARG1 or expressing a catalytically inactive mutant did not show obvious defects in protein biosynthesis (Supplementary Fig. S6), in agreement with the lack of effects on cell proliferation in HeLa cells.

### EGFP-TARG1 accumulates in transcriptionally active nucleoli independent of ADP-ribose binding

**Figure 3:**
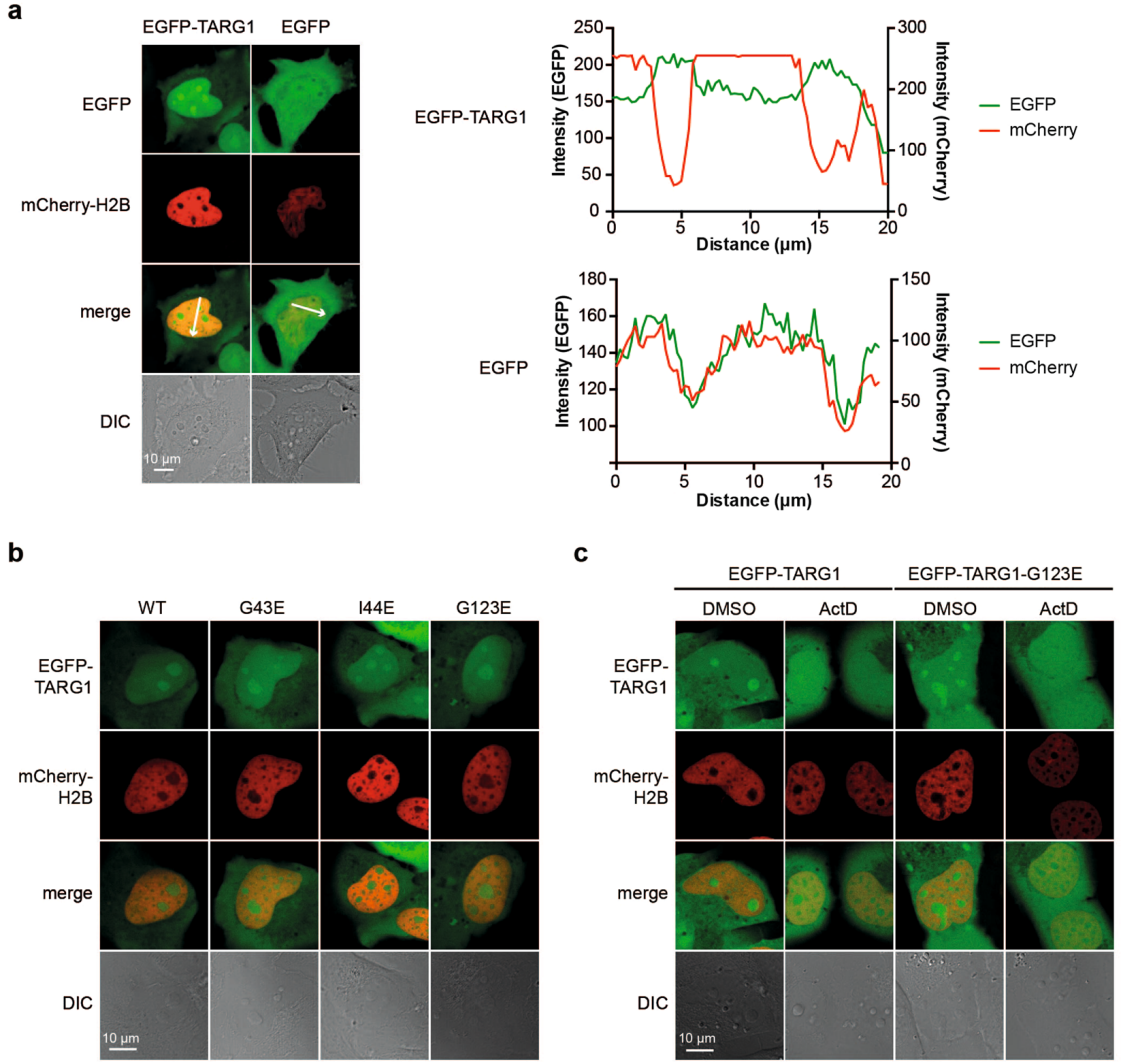
EGFP-TARG1 localizes to transcriptionally active nucleoli independent of ADP-ribosylation. (**a**) Live cell imaging of U2OS cells transiently expressing EGFP-TARG1 or EGFP together with mCherry-H2B. Intensity profiles for EGFP and mCherry fluorescence signals are displayed that were measured along the arrows depicted in the merge pictures. DIC: differential interference contrast. (**b**) Live cell imaging of U2OS cells transiently expressing EGFP-TARG1 wildtype (WT), G43E, I44E or G123E together with mCherry-H2B. For intensity profiles, see Supplementary Figures S7. (**c**) U2OS cells transiently expressing EGFP-TARG1 or EGFP-TARG1-G123E together with mCherry-H2B were treated with 10 ng/ml actinomycin D (ActD) or DMSO for 90 min. Subcellular localization of EGFP-TARG1 (wildtype or G123E) was analyzed by confocal microscopy in living cells.

EGFP-TARG1 was reported to be predominantly nuclear^9^, and our proteomics data set contained a high number of nuclear and in particular nucleolar proteins, also reflected by GO analyses for cellular component (Supplementary Table S3). Therefore, we analyzed the sub-cellular localization of TARG1 in greater detail. EGFP-TARG1 expressed in U2OS cells accumulated in nucleoli in living cells, while EGFP alone was excluded from nucleoli (Fig. 3a). mCherry-tagged Histone H2B was used to define the nucleoplasm^28^.

TARG1 is recruited to PAR chains during DNA damage and PAR and ARTD1/2 are found enriched in nucleoli in non-stressed cells^2,9,29–33^. Therefore, we asked whether nucleolar accumulation of TARG1 is dependent on its ability to interact with ADPr. All three ADPr binding-deficient TARG1 mutants accumulated in nucleoli in U2OS cells (Fig. 3b, Supplementary Fig. S7). Furthermore, treatment of U2OS cells with olaparib did not change the sub-cellular distribution of EGFP-TARG1 (see also below), together indicating that nucleolar localization of TARG1 is not dependent on nucleolar PAR.

The nucleolus is involved in the cellular stress response, in line with rapid changes of the nucleolar proteome upon stress^34^. Inhibition of rRNA transcription by actinomycin D results in nucleolar disruption and release of nucleolar proteins, including ribosomal proteins, into the nucleoplasm^34^. Actinomycin D treatment, which at low concentration inhibits primarily RNA polymerase I, abrogated nucleolar accumulation of EGFP-TARG1 (Fig. 3c). The same was observed for EGFP-TARG1-G123E, indicating that loss of nucleolar accumulation of TARG1 upon actinomycin D treatment was PARylation-independent (Fig. 3c). Thus, TARG1 is recruited into transcriptionally active nucleoli independent of ADPr binding.

### EGFP-TARG1 is rapidly exchanging between the nuclear and the nucleolar compartment

**Figure 4:**
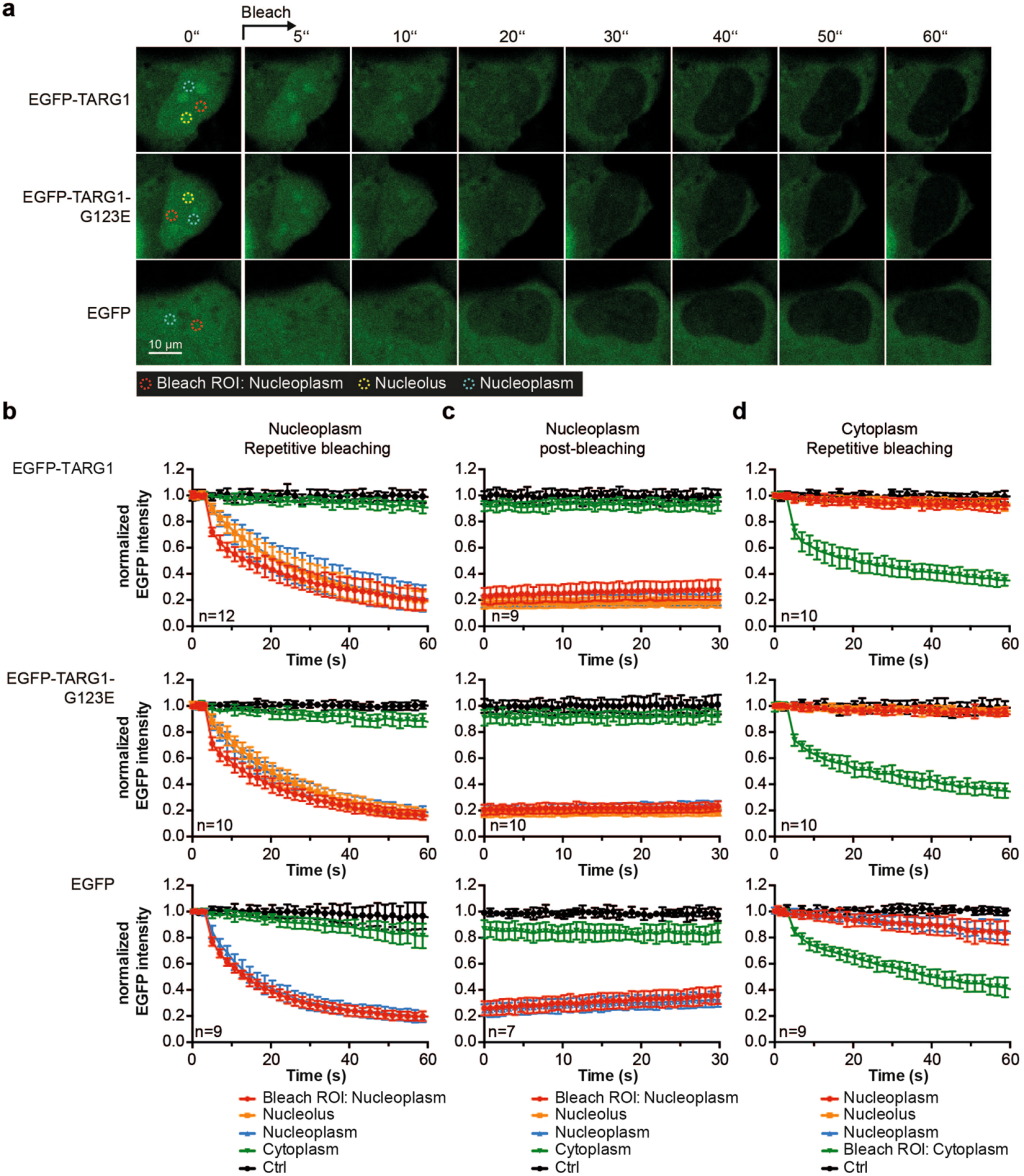
EGFP-TARG1 rapidly shuttles between the nucleoplasmic and the nucleolar compartment. (**a**) Fluorescence loss in photo-bleaching (FLIP) experiments in U2OS cells transiently expressing EGFP-TARG1, EGFP-TARG1-G123E or EGFP. mCherry-H2B was co-expressed to define the nucleoplasm (not shown). Representative images are shown. (**b–d**) EGFP fluorescence intensities of EGFP-TARG1 (upper panels), EGFP-TARG1-G123E (middle panels) and EGFP (lower panels) were measured in each ROI during photo-bleaching of nucleoplasmic EGFP (**b**), after total photo-bleaching of nucleoplasmic EGFP (**c**) or during photo-bleaching of cytoplasmic EGFP (**d**). Fluorescence intensities were normalized to the mean EGFP fluorescence intensity in the same ROI before bleaching and are expressed as mean ± SD of 7–12 cells as indicated.

The nucleolar compartment consists of highly dynamic protein-protein and protein-nucleic acid interactions and nucleolar components typically continuously exchange with the surrounding nucleoplasm, important for quickly responding to cellular stress^35–38^. Therefore, we assessed the exchange of EGFP-TARG1 between the nucleoplasm and nucleoli using fluorescence loss in photo-bleaching (FLIP) experiments in U2OS cells. Several regions of interest (ROIs) were defined, one for bleaching and one for measuring were placed in the nucleoplasm, one in a nucleolus, one in the cytoplasm, and a control ROI in a neighboring cell to account for changes during the measurements. After acquisition of five control images, one nucleoplasmic ROI was exposed to repetitive photo-bleaching. After each bleach pulse imaging scans were performed and mean EGFP intensities were determined in all ROIs. EGFP fluorescence intensities in control regions remained constant (Fig. 4b–d), indicating that the effect of bleaching during image acquisition between the bleach pulses was negligible. EGFP intensities in each ROI were normalized to the mean EGFP intensity in each ROI before bleaching (Fig. 4b–d).

Nucleoplasmic bleaching in EGFP-TARG1 expressing cells resulted in depletion of nucleoplasmic EGFP fluorescence to ∼20% within 60 s (Fig. 4a and b, upper panel), suggesting a high mobility of EGFP-TARG1 within the nucleoplasm. Normalized nucleolar EGFP fluorescence decreased together with nucleoplasmic EGFP fluorescence, indicating that EGFP-TARG1 exchanged rapidly between nucleoli and the nucleoplasm (Fig. 4b, upper panel). In contrast, EGFP fluorescence intensities in the cytoplasm decreased only slightly during 1 min of nuclear photo-bleaching (Fig. 4b, upper panel). As bleaching of a nuclear region can be accompanied by bleaching of cytoplasmic EGFP fusion proteins above and below the nucleus, a reciprocal experiment was performed, in which cytoplasmic EGFP was bleached. No marked decrease in nuclear/nucleolar EGFP intensity was observed (Fig. 4d, upper panel), altogether indicating that within 1 min no substantial nucleo-cytoplasmic EGFP-TARG1 shuttling occurred.

In U2OS cells expressing EGFP alone, nucleoplasmic EGFP fluorescence was depleted to ∼19% within 60 s, which is comparable to what was observed for EGFP-TARG1 (Fig. 4a and b, bottom panel). However, in contrast to EGFP-TARG1, a clear nucleo-cytoplasmic exchange of EGFP was observed during this time frame (Fig. 4b, bottom panel and Fig. 4d, bottom panel). Thus, EGFP but not EGFP-TARG1 exchanged rapidly between the cytosol and the nucleus.

We noticed that EGFP-TARG1 accumulated, albeit weakly, in the bleach ROI post-bleaching but not in a second ROI in the same nucleus (Fig. 4c, upper panel). Because EGFP-TARG1 is recruited to sites of DNA damage^9^ and photo-bleaching of fluorophores can be accompanied by the production of reactive oxygen species^39^, we addressed whether PARylation was important for this effect. We performed FLIP experiments with EGFP-TARG1-G123E, which is ADPr binding-deficient and is not recruited to DNA damage sites^9^. In EGFP-TARG1-G123E expressing cells, no accumulation of signal in the bleach ROI was observed (Fig. 4c, middle panel), indicating that EGFP-TARG1 is indeed to some extent recruited into the bleached region by ADP-ribosylation. However, EGFP-TARG1-G123E displayed an overall nuclear mobility and nucleoplasmic-nucleolar exchange similar to EGFP-TARG1 (Fig. 4a and b, middle panel). Thus, the influence of photo-bleaching-induced PARylation in the bleach ROI is negligible in our experiments.

### Modulation of nucleolar TARG1 localization by H_2_O_2_-induced DNA damage stress

**Figure 5:**
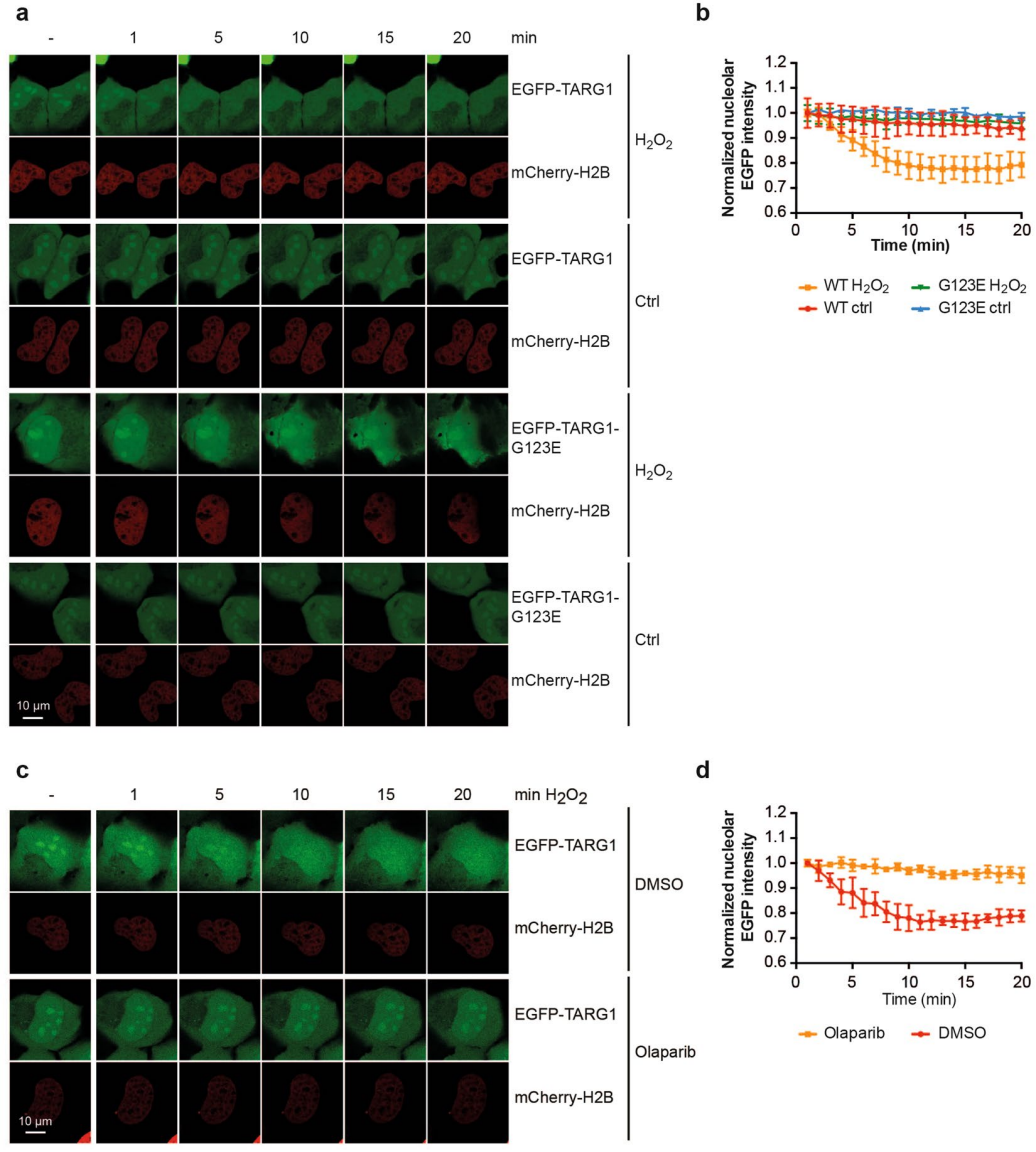
Nucleolar accumulation of TARG1 is lost upon H_2_O_2_-treatment. (**a**) U2OS cells transiently expressing EGFP-TARG1 or EGFP-TARG1-G123E together with mCherry-H2B were or were not (ctrl) treated with 1 mM H_2_O_2_. Subcellular localization of EGFP-TARG1 (wildtype/G123E) before (−) and during the first 20 min after treatment was analyzed in living cells by confocal microscopy. A representative experiment is depicted. (**b**) Quantifications of nucleolar EGFP intensities from cells imaged during the experiment depicted in panel A are given. Nucleolar EGFP-intensities were measured using ImageJ, normalized to nuclear EGFP-intensities at each time point and to the normalized nucleolar EGFP intensity 1 min after treatment (mean ± SD of at least 3 cells). (**c**) U2OS cells transiently expressing EGFP-TARG1 wildtype together with mCherry-H2B were treated with DMSO or 10 µM olaparib for 2 h before treatment with 1 mM H_2_O_2_. Subcellular localization of EGFP-TARG1 before (−) and during the first 20 min after treatment was analyzed in living cells by confocal microscopy. A representative experiment is depicted. (**d**) Quantifications of nucleolar EGFP intensities from cells imaged during the experiment depicted in panel c are given. Intensities were calculated as in panel b.

Next we asked whether modulation of nuclear PAR levels would affect the subnuclear localization of TARG1. Hydrogen peroxide (H_2_O_2_), commonly used to induce oxidative DNA damage, activates ARTD1 and results in PARylation of ARTD1 and chromatin-associated proteins. PAR levels peak within 10–15 min after treatment and are degraded quickly thereafter by PARG^40^. In response to H_2_O_2_ treatment, EGFP-TARG1 was rapidly lost from nucleoli, but did not affect the localization of EGFP-TARG1-G123E (Fig. 5a and b). Olaparib prevented H_2_O_2_-induced re-location of TARG1, in agreement with a PARylation-dependent accumulation of EGFP-TARG1 in the nucleoplasm (Fig. 5c and d).

### TARG1 interacts with RNA

**Figure 6:**
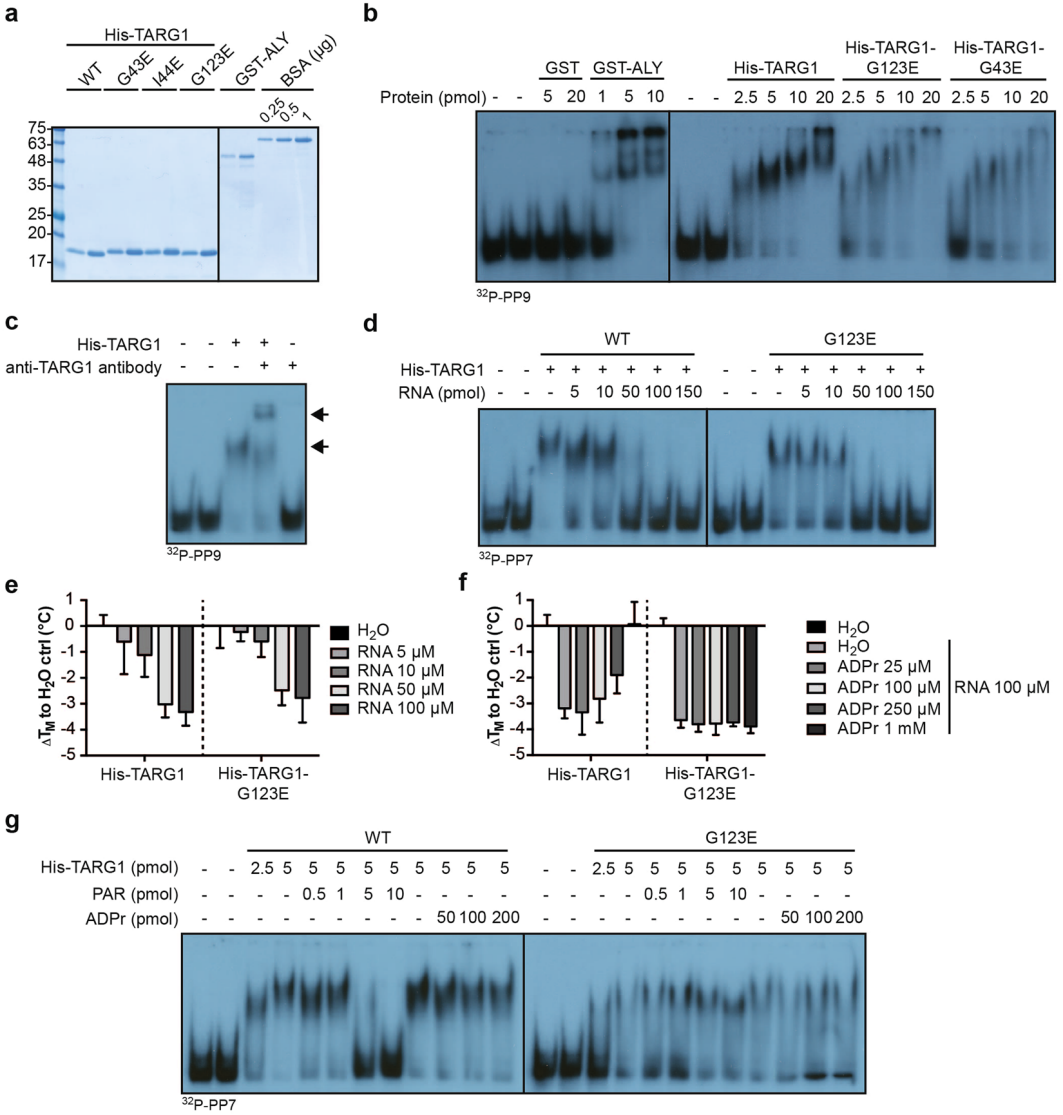
TARG1 is an RNA binding macrodomain. (**a**) TARG1 wildtype, -G43E, -I44E and -G123E were expressed as N-terminal His_6_-tag fusion proteins in bacteria and purified by immobilized metal affinity chromatography (IMAC). ALY, fused to an N-terminal glutathione S-transferase (GST)-tag, was expressed in bacteria and purified by glutathione affinity chromatography. Aliquots of the eluates, together with a bovine serum albumin (BSA) standard, were separated on an SDS-gel and stained with Coomassie blue. (**b**) Electrophoretic mobility shift assay (EMSA) using RNA Pentaprobe oligonucleotides^41^. Purified ^32^P-labeled Pentaprobe 9 (^32^P-PP9) was incubated with the indicated amounts of purified GST, GST-ALY, His-TARG1 wildtype (WT), -G123E or -G43E (2.5–20 pmol corresponding to 83.3–666 nM, respectively). Free RNA and RNA-protein complexes were separated on native 7% poly-acrylamide gels. Mobility shifts were analyzed by auto-radiography. (**c**) EMSA performed as described in panel b with ^32^P-PP9 and His-TARG1 (5 pmol, 0.16 µM), His-TARG1 in complex with a polyclonal antibody raised against TARG1 (Eurogentec) or with antibody alone. (**d**) EMSA of ^32^P-PP7 that was incubated with constant amounts of His-TARG1 (5 pmol, 0.16 µM) and increasing amounts of cellular RNA (5–150 pmol, corresponding to 0.16–5 µM, calculated per nucleotide). (**e**) Thermal shift assay of 2 µM His-TARG1 WT or -G123E together with increasing amounts of cellular RNA (5–100 µM, calculated per nucleotide). Melting temperatures were determined according to^25^ and are presented as ΔT_M_ to H_2_O control (ctrl; mean ± SD of 3 experiments). (**f**) Thermal shift assay of 2 µM His-TARG1 WT or -G123E in the presence of constant amounts of cellular RNA (100 µM, calculated per nucleotide) together with increasing amounts of ADPr. Melting temperatures are expressed as ΔT_M_ to H_2_O control (ctrl; mean ± SD of 3 experiments). (**g**) EMSA of ^32^P-PP7 that was incubated with constant amounts of His-TARG1 WT or -G123E (5 pmol, 0.16 µM) and increasing amounts of purified PAR (0.5–10 pmol, calculated per ADPr unit, corresponding to 0.016–0.33 µM) or ADPr (50–200 pmol, corresponding to 1.66–6.66 µM).

Localization of proteins in nucleoli is often mediated by interaction with nucleolar RNA or by binding to nucleolar hub proteins such as NCL or nucleophosmin^35^. Therefore, we asked whether TARG1 has RNA binding activity. In an RNA electrophoretic mobility shift assay (EMSA) His-tagged TARG1 bound dose-dependently to ^32^P-labeled ssRNA Pentaprobes (Fig. 6a and b)^41^. The Pentaprobe plasmid library encodes six overlapping double stranded 100 bp long oligonucleotides that in total contain every possible 5 bp sequence motif, from which 12 different ssRNA molecules can be *in vitro* transcribed (forward and reverse)^41^. RNA binding activity of GST-ALY served as positive control (Fig. 6a and b)^42,43^, while GST alone did not bind (Fig. 6a and b). The specificity of the TARG1-RNA complex was documented by the induction of a supershift with a TARG1-specific antibody (Fig. 6c). TARG1 interacted equally well with several different Pentaprobe constructs (Fig. 6b and d), indicating that TARG1 binding to RNA is likely independent of a specific sequence. Unlabeled RNA isolated from cultured cells competed with TARG1 binding to the Pentaprobes (Fig. 6d).

To determine whether binding of RNA by TARG1 involves interactions with the ADPr binding site, we analyzed RNA binding by His-TARG1-G123E and -G43E (Fig. 6a). Both mutants bound to RNA in the same concentration range as His-TARG1 (Fig. 6b). Similarly, ADPr did not prevent RNA binding by TARG1 (Fig. 6g), indicating that the interaction with RNA is not or not exclusively based on binding in the ADPr binding pocket. Also, RNA did not increase thermal stability of TARG1 (Fig. 6e), as in the case of ADPr (Supplementary Fig. S1d). Instead, addition of increasing amounts of RNA to TARG1 reduced its thermal stability (Fig. 6e). When titrating ADPr into an RNA-TARG1 mixture in the thermal shift assay, the negative thermal shift induced by RNA was reversed in a dose-dependent manner for TARG1, but not for TARG1-G123E (Fig. 6f).

The TARG1-associated proteome changed dramatically in response to PARylation. We reasoned that this drastic change could be caused by a switch from RNA to PAR binding of TARG1. We therefore tested whether PAR chains were able to compete with RNA for binding to TARG1. Indeed, titration of PAR into the RNA binding assay efficiently reversed the band shift induced by His-TARG1 but poorly by His-TARG1-G123E (Fig. 6g). These data, together with the inability of ADPr to compete, are in agreement with a model, in which TARG1 binds RNA via positively charged patches on its surface but not via its ADPr binding pocket. In contrast, PAR occupies the ADPr binding pocket of TARG1 and at the same time interacts very likely with the positively charged patches on the surface of TARG1. These findings support the notion that TARG1 has a higher affinity to PAR than to RNA.

## Discussion

**Figure 7:**
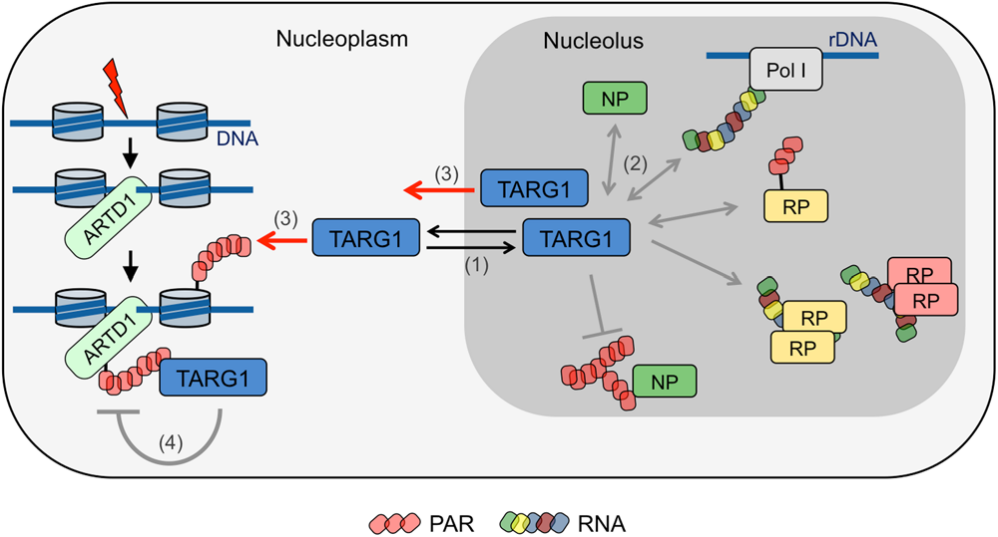
Model of a dual function of TARG1 in both RNA- and PAR-regulated cellular processes. Under steady-state conditions, TARG1 accumulates in nucleoli but constantly shuttles between nucleoli and the nucleoplasm (1). Accumulation of TARG1 in nucleoli is mediated by direct interaction with ribosomal RNA or proteins (RP) or other nucleolar proteins (NP; 2). PARylation modulates the localization and interactome of TARG1. TARG1 re-locates to the nucleoplasm upon DNA damage-induced ARTD1/2-dependent PARylation (3). In nucleoli, TARG1 may contribute to ribosome biogenesis, e.g., by counteracting PARylation of RPs or controlling nucleolar PAR scaffolds. DNA damage-induced PARylation might serve to sequester TARG1 to the nucleoplasm to regulate PAR turnover at DNA damage sites (4). Hypothetical interactions are marked by grey arrows.

We have identified two different interactomes of TARG1, which are dependent on the presence or absence of PAR. In the absence of ARTD1/2 activation and PAR formation, the TARG1 interactome is strongly directed toward ribosomal proteins and RNA-binding proteins (RBPs) that function in diverse RNA-associated cellular processes. Furthermore, we observed that TARG1 is a nuclear protein that shuttles rapidly between the nucleoplasm and nucleoli. The distribution between these two compartments is regulated by PARylation, i.e. TARG1 is mainly nucleolar in the absence of PAR, while it accumulates in the nucleoplasm in response to PAR formation. We also discovered that TARG1 interacts with RNA, which together with binding to ribosomal proteins is suggested to control its nucleolar localization (Fig. 7).

The binding to ribosomal proteins and RBPs is consistent with a strong connection between RNA biology and ADP-ribosylation, which has been repeatedly found in proteomics screens for proteins associated with stress-induced ADP-ribosylation^23,24,44–50^. These screens commonly revealed that a high number of RNA processing factors are ADP-ribosylated or are PAR binding proteins^44,45^. So far, it is unclear which of the identified proteins interact directly with TARG1. In addition to direct protein-protein binding, we assume that some of the interactions are mediated by the ability of TARG1 to associate with RNA (Fig. 6). This seems likely as a number of proteins were identified that are part of multi-protein and ribonucleoprotein complexes such as ribosomes. In contrast, when PARylation was not inhibited the interactome was dominated by DNA repair proteins of various processes, most of which have been shown to be PARylated or interacting with PAR^23,24,47,51–54^. This observation is consistent with the finding that TARG1 is recruited to sites of DNA damage in an ARTD1-dependent manner^9^. This efficient recruitment is also in agreement with the strong enrichment of ARTD1 in the TARG1 interactome in the absence of olaparib (Fig. 1).

Unlike the PAR-dependent recruitment of TARG1 to DNA damage sites^9^, its nucleolar localization and its shuttling between nucleolus and nucleoplasm is independent of ADPr binding. This might seem surprising, because ADP-ribosylation has been associated with nucleolar functions in a number of studies^29,44,55–57^. PAR, ARTD1 and ARTD2 can be found enriched in nucleoli under non-stress conditions and several nucleolar proteins are ADP-ribosylated and/or interact with ARTD1, ARTD2 or PAR^29,32,33,55^. Interference with PAR metabolism in *Drosophila* results in mislocalization of nucleolar proteins and disintegration of nucleoli^55^. We did not observe fragmentation or other abnormalities of nucleoli in cells with *OARD1* knock-out or TARG1 overexpression. In *Drosophila Parp* and *Parg* mutants, rRNA maturation defects have been observed, accompanied by a drop in cytoplasmic polysomes^55^. It was proposed that nucleolar PAR serves as a scaffold to recruit rRNA processing factors into nucleoli, which is required for accurate ribosome biogenesis^55^. PAR chains formed at DNA damage sites are capable of forming liquid phase structures together with proteins that contain low complexity domains^58^. Thus, PAR might also contribute to the liquid phase structure of nucleoli, which are formed around rDNA repeats in an incremental manner by multivalent interactions between rRNA transcripts and nucleolar proteins^2,35,59–61^. TARG1 inhibits ARTD1 *in vitro* and might be able to remove complete PAR chains from automodified ARTD1^9^. It remains however unknown whether TARG1 is able to modulate PARylation levels by either of these mechanisms in cells. Whether TARG1 is needed for nucleolar integrity by modulating nucleolar PARylation or by other means remains to be investigated.

Nucleolar localization of TARG1 could be mediated by direct interaction of TARG1 with nucleolar RNA. This is supported by the fact that two ADPr binding-deficient TARG1 mutants that retain RNA binding ability still localize to nucleoli and that TARG1 wildtype and mutants only locate to transcriptionally active nucleoli. Actinomycin D treatment induces broad changes in the nucleolar proteome^62^. Especially ribosomal proteins and ribosome biogenesis factors depend on the presence of rRNA for nucleolar localization and leave nucleoli upon RNA polymerase I inhibition^34,62^. Thus, in addition to interaction with rRNA, the possibility exists that the actinomycin D-induced loss of nucleolar accumulation of TARG1 is caused by more indirect mechanisms than the loss of direct interaction between TARG1 and nucleolar RNA.

We note that TARG1 was not identified to reside in nucleoli in a number of proteomics studies that assessed the composition of the nucleolar proteome. This suggests that the association of TARG1 with nucleoli is not sufficiently stable and thus might be lost during purification of nucleoli, especially if purifications are performed in the absence of ARTD1/2 inhibitors^62–64^.

Several reasons encouraged us to address a potential RNA binding activity of TARG1. These included the localization of the protein to nucleoli, the interaction with ribosomes and RBPs, and the finding that several macrodomains including the macrodomain of CHIKV-nsP3 and MacroD1 interact with RNA^14,15^. Our study provides an initial characterization of the RNA binding activity of TARG1. We found TARG1 to interact with different RNA molecules in EMSAs, indicating that the binding is independent of the RNA sequence. The competition experiments suggested that RNA binding does not rely on the ADPr binding pocket. PAR efficiently competed with RNA for binding to TARG1, but not for binding to TARG1-G123E. We conclude that PAR occupies the same regions in TARG1 as RNA, explaining the competitive binding mode. The requirement for an intact ADPr binding pocket for efficient competition suggests that PAR binding to this pocket is substantial. This is consistent with the roughly 10-fold more efficient competition of TARG1 RNA binding by PAR compared to RNA and by the inefficient competition by ADPr (Fig. 6). Thus, RNA binding occurs probably in the vicinity of the ADPr binding pocket of TARG1 and the efficient competition by PAR requires interaction in this pocket and at surface sites that also interact with RNA. The latter is supported by the presence of basic surface patches close to the ADPr binding pocket in TARG1^9^, which could mediate unspecific interaction with the negatively charged RNA backbone and also stabilize interaction with PAR, as suggested earlier^14,15^.

RNA induced a dose-dependent reduction of the TARG1 melting temperature (Fig. 6). The decrease in T_M_ of His-TARG1 upon incubation with RNA is likely to reflect the direct interaction of RNA with the protein, although T_M_ decreases in the thermal shift assays have also been attributed to indirect effects^65^. Destabilization of TARG1 by RNA was antagonized by ADPr. Of surprise was the high concentration of ADPr necessary for the stabilization effect, indicating that the affinity for ADPr is rather low. This is consistent with a K_i_ value of 119 µM for ADPr in OA-ADPr deacetylation reactions^12^, but less compatible with the K_d_ of 8.4 µM for ADPr^9^. Together with the ADPr competition in EMSAs, we conclude that ADPr and RNA are able to interact with TARG1 simultaneously.

What is the functional consequence of TARG1 binding to RNA and ribosomal proteins? How does this relate to the role of TARG1 during DNA damage-induced PARylation? So far, it is not clear whether TARG1 directly regulates ribosome biogenesis. According to our proteomics data, TARG1 interacts with a variety of ribosomal proteins and ribosome biogenesis factors, but evidence for a possible role of TARG1 in particular steps of ribosome biogenesis is missing presently. Based on the recruitment of TARG1 to sites of DNA damage and a decreased resistance to DNA damaging agents of TARG1 knock-down cells, TARG1 was proposed to act as a DNA damage response factor^9^, a finding that in our experimental set-up could not be reproduced (Supplementary Fig. S5). However, several RBPs and ribosomal proteins have been described as direct substrates of ADP-ribosylation during genotoxic stress^44,66^. Some of these ribosomal proteins are important for the interaction of the 40S and 60S subunits^66,67^. It was proposed that ARTD1-dependent ADP-ribosylation of RPL24 at E106 and RPS8 at E89 might interfere with the formation of inter-subunit bridges formed between RPL24 and RPS6 and between RPS8 and the 28S rRNA, thereby regulating ribosome assembly^66^. These observations were made in a breast cancer cell line, but not in several other lines. Nevertheless, it offers the intriguing possibility that ADP-ribosylation of these proteins regulates ribosome assembly. Whether TARG1 can reverse these modifications and thereby modulate ribosome biogenesis remains to be determined. The maintenance of nucleolar PAR levels seems to promote nucleolus formation and ribosome biogenesis in *Drosophila*^55^. Whether this is also occurring in mammalian cells needs to be demonstrated. Nevertheless, the role of PARylation in nucleolar functions might be more complex than anticipated. PARylation might affect nucleolar functions by several mechanisms, acting either as a scaffold important for the recruitment of ribosome biogenesis factors and at the same time as a PTM of ribosomal proteins, interfering with ribosome assembly. This might explain why the effects observed in *OARD1* knock-out cells are rather weak. The presence of MacroD1 and/or MacroD2 might also compensate sufficiently for the lack of TARG1 and thus allow proliferation, at least in HeLa cells.

DNA damage-induced PARylation sequesters TARG1 in the nucleoplasm, where it may contribute to PAR turnover at DNA damage sites (Fig. 7). It is tempting to speculate that this sequestration may prevent TARG1 from executing a so far unidentified nucleolar function, thereby contributing to the cellular stress response. The loss of TARG1 in humans correlates with a severe neurodegeneration phenotype^9^, which might be due to a chronic, suboptimal ribosome production. Thus, it will now be interesting to address the role of TARG1 in animal models, in which long-term consequences can be studied.

## Materials and Methods

### Plasmids and oligonucleotides

The *OARD1* coding sequence was amplified from human cDNA using Gateway (Invitrogen)-compatible primers and cloned into pDONR/Zeo (Invitrogen). GW-p-N-TAP-C6orf130 was generated by Gateway recombination between pDONR/Zeo-C6orf130 and a Gateway-compatible pcDNA5/FRT/TO-N-TAP vector^20^.

A sequence-optimized TARG1/C6orf130 fragment was amplified from pNic28-BSA4-C6orf130 (kindly provided by H. Schüler, Karolinska Institute, Stockholm) using Gateway-compatible primers and recombination into pDONR/Zeo. This pDONR/Zeo-C6orf130 clone was used for gateway cloning of the *OARD1* coding sequence into pDEST17 (Invitrogen), pcDNA5/FRT/TO (Invitrogen), GW-pEGFP^68^, pcDNA3-FLAG, pLKO-T-REX-HA-DEST-Neo (kindly provided by F. Stegmeier, Novartis). For generation of pcDNA5/FRT/TO-EGFP-TARG1 plasmids, the EGFP-TARG1 fragment from GW-pEGFP-TARG1 was amplified using primers containing *Bam*HI restriction sites and cloned into pcDNA5/FRT/TO. Mutants were generated by site-directed mutagenesis. The mCherry-H2B plasmid was a kind gift from E. Ferrando-May (Bioimaging Center, University of Konstanz). The plasmid for bacterial expression of GST-ARTD10(818–1025) has been described^20^. pGEX-4T1-ALY served as a plasmid for bacterial expression of GST-ALY. pcDNA3.1-based Pentaprobe plasmids were a gift from J. Mackay^41^.

For the generation of Flp-In™ T-REx™ cell lines with inducible expression of shRNA constructs, a modified pcDNA5/FRT/TO vector was constructed: The H1 promoter from pSUPER^69^ was modified with Tet regulator sequences. The fragment containing the modified H1 promoter and the shRNA cloning site was cloned into pcDNA5/FRT/TO, replacing the CMV/TetO_2_ promoter. A second *Bgl*II site in the resulting pcDNA5 vector was mutated. The resulting pcDNA5/FRT/TO-Super-Tet vector was digested with *Hind*III and *Bgl*II and ligated with annealed pairs of the oligonucleotides shTARG1_ORF_fwd (gatcgagagatgggcgatatatacgaatatatatcgcccatctctc) and shTARG1_ORF_rev (agctgagagatgggcgatatatattcgtatatatcgcccatctctc) or shCtrl_fwd (gatcagaagagtttagaggcaatcgaaattgcctctaaactcttc) and shCtrl_rev (agctgaagagtttagaggcaatttcgattgcctctaaactcttct). shRNA sequences (underlined) were taken from^9^.

For the generation of CRISPR-Cas9(D10A) knock-out constructs oligonucleotides defining the gRNA sequences were annealed in pairs and cloned into a modified pX335 vector (Addgene 42335) containing a GFP-puromycin selection cassette^70–72^ and verified by sequencing: (1) forward: caccccaattacagcatgtgtaaa, reverse: aaactttacacatgctgtaattgg (2) foward: cacccagattggaggaaggggtg, reverse: aaacgcaccccttcctccaatctg (3) foward: caccgctctctgctaagcaggctg, reverse: aaaccagcctgcttagcagagagc (4) forward: caccagcaccctctgcttgaagct, reverse: aaacagcttcaagcagagggtgc.

### Cell lines and cell culture

All cell lines were cultivated in DMEM supplemented with 10% heat-inactivated fetal calf serum (FCS) and 1% penicillin/streptomycin at 37 °C in 5% CO_2_ in a humidified atmosphere. Transfections were performed using calcium phosphate precipitation.

HeLa Flp-In™ T-REx™ (kindly provided by Stephen Taylor, University of Manchester) and HEK293 Flp-In™ T-REx™ cells (Invitrogen) were generated according to manufacturer’s instructions with the plasmids pcDNA5/FRT/TO, pcDNA5/FRT/TO-TARG1, pcDNA5/FRT/TO-TARG1 G123E, GW-pN-TAP-C6orf130, pcDNA5/FRT/TO-Super-Tet-shOARD1, pcDNA5/FRT/TO-Super-Tet-shCtrl and selected with hygromycin B and blasticidin S. HEK293 Flp-In™ T-REx™ N-TAP cells were described previously^20^.

HeLa *OARD1*^−/−^ cell clones were generated by transfection of HeLa cells with equal amounts of four different pX335 plasmids encoding the four different gRNAs for 24 h, followed by selection of transfected cells with 1 µg/ml puromycin for 24 h. Single cell clones were picked after ∼14 days and were expanded. Genomic DNA was isolated from each clone using the High Pure PCR Template Preparation Kit (Roche) and used as a template in genotyping PCR reactions using oligonucleotides Ex4-fwd (tgggttgtgaggaaacatga) and Ex4-rev (gccatcactggactggagtt) for amplification of the wildtype allele and Ex1_fwd (tggttgtacagggcaatcag) and Int5-rev (ttgcaacaccctggtaagaa) for identification of the deletion.

HeLa *OARD1*^−/−^ HA-TARG1 and HeLa *OARD1*^−/−^ HA-TARG1-G43E cells were generated by lentiviral transduction of HeLa *OARD1*^−/−^ cells with pLKO-TREX-HA-TARG1 plasmids and were selected with G-418.

### Antibodies and reagents

Polyclonal rabbit anti-TARG1 was raised against two TARG1 peptides (1) aa 138–152 (EVFEATDIKITVYTL) and (2) aa 119–134 (RIGCGLDRLQWENVSA; Eurogentec). Monoclonal antibodies were generated against His_6_-TARG1 (full-length, gift from I. Ahel, University of Oxford): 3A5 (rat), 31F6 (mouse), 6F11 (rat), 28E9 (mouse) and 26E4 (mouse) (E. Kremmer, R. Feederle). Additional antibodies used were as follows: anti-SSRP1 (E1Y8D, Cell Signaling Technology), XRCC1 (#2735BC, Cell Signaling Technology), anti-CHD1L (E1I8C, Cell Signaling Technology), anti-PARP1 (1835238, Roche), anti-KU80 (C48E7, Cell Signaling Technology), anti-NCL (4E2, abcam), anti-RPL7a (E109, Cell Signaling Technology), anti-USP10 (D7A5, Cell Signaling Technology), anti-α-Tubulin (B512, Sigma-Aldrich), anti-puromycin (12D10, Merck Millipore), peroxidase-conjugated goat anti-rabbit IgG (H + L), peroxidase-conjugated goat anti-mouse IgG (H + L), peroxidase-conjugated goat anti-rat IgG + IgM (H + L) (Jackson Immunoresearch). Oligonucleotides were purchased from Sigma-Aldrich. The following reagents were used: β-NAD^+^ (Sigma-Aldrich), ^32^P-NAD^+^ (Perkin Elmer), α-^32^P-UTP (Hartmann Analytic), adenosine 5′ diphosphoribose sodium-salt (Sigma-Aldrich), poly-ADP-ribose (4336–100–01, Trevigen), olaparib (Sellekchem), protease inhibitor cocktail P8340 (Sigma-Aldrich), RNase inhibitor, murine (M0314, NEB), AcTEV protease (Invitrogen), glutathione agarose (Pierce™), TALON® metal affinity resin (Clontech), IgG Sepharose® 6 Fast Flow (GE Healthcare), Calmodulin Sepharose® 4B (GE Healthcare), anti-FLAG® M2 affinity gel (Sigma-Aldrich), actinomycin D (Sigma-Aldrich), puromycin (AppliChem), hygromycin B (Invivogen), blasticidin S (Invivogen), G-418 (Invivogen), DMSO (AppliChem), H_2_O_2_ (Merck KGaA), cell proliferation reagent WST-1 (Roche), bovine serum albumin (BSA; AppliChem), etoposide (Biomol), doxorubicin (Sigma-Aldrich), hydroxyurea (Sigma-Aldrich), SYPRO® orange (ThermoFisher Scientific), doxycycline (Sigma-Aldrich).

### Tandem affinity purification

Large-scale purification of TAP-TARG1-containing protein complexes was performed as described previously^20^ with a few modifications: 5 × 10^7^ cells were trypsinized and transferred to spinner flasks in 250 ml non-selective cell culture medium. Cells were cultivated under constant stirring at 37 °C, 5% CO_2_ and diluted with fresh medium every 24–48 h to a final culture volume of 1 l. Cells were treated with 1 µg/ml doxycycline for 14 h to induce expression of TAP fusion proteins. All harvesting, centrifugation and purification steps were performed at 4 °C or on ice. Cells were pelleted at 200 × g and washed once with ice-cold PBS. The cells were pelleted again, were resuspended in 20 ml lysis buffer (50 mM Tris pH 7.5, 150 mM NaCl, 1 mM EDTA, 10% (v/v) glycerol, 1% (v/v) Nonidet P-40, 1 mM DTT, 100 μM sodium vanadate, 1x protease inhibitor cocktail, ±10 µM olaparib) and lysed for 30 min under permanent agitation. Lysates were centrifuged at 15,000 × g for 20 min. The supernatant was incubated with 160 μl equilibrated IgG Sepharose for 1 h under permanent agitation. Beads were pelleted at 500 × g for 3 min and washed three times with TEV buffer (50 mM Tris pH 7.5, 150 mM NaCl, 0.5 mM EDTA, 1 mM DTT). The beads were pelleted and TEV cleavage was performed in 300 µl TEV buffer with 3 µl (30 U) of TEV protease for 3 h under permanent agitation. The bead supernatants were then transferred to a fresh tube. The IgG Sepharose pellet was resuspended in three volumes of CaM binding buffer (10 mM Tris pH 7.5, 150 mM NaCl, 0.2% (v/v) Nonidet P-40, 1 mM magnesium acetate, 2 mM CaCl_2_, 1 mM imidazole, 10 mM β-mercaptoethanol) and pelleted again. The supernatant and the supernatant of the TEV cleavage were pooled for the subsequent CBP pulldown, which was performed after addition of 1/200 volume of 1 M CaCl_2_ to the supernatants with 50 µl equilibrated Calmodulin Sepharose for 90 min under permanent agitation. The beads were washed three times with CaM wash buffer (50 mM ammonium bicarbonate pH 8.0, 75 mM NaCl, 1 mM magnesium acetate, 1 mM imidazole, 2 mM CaCl_2_). Residual buffer was removed carefully from the pellet and dry pellets were stored at −80 °C until mass spectrometry analysis.

### Mass spectrometry analysis and data analysis

The dried pellets were processed for MS-analysis as described previously^73^. In brief, the dried beads from the TAP-purification were digested for 1 hour at room temperature in 2 M urea, 50 mM Tris-HCl pH 7.5 and 5 μg/mL Trypsin, followed by two washes with 2 M urea, 50 mM Tris-HCl pH 7.5 and 1 mM DTT. The pooled supernatants were left to digest o/n at RT. After iodoacetamide modification and acidification of the samples, the peptides were desalted using homemade C18-tips and lyophilized. Peptides were analyzed on a Q-Exactive mass spectrometer connected to an Ultimate Ultra3000 chromatography system incorporating an autosampler (both Thermo Scientific). Proteolytic peptides for each sample (5 μL) were applied to a home-made column (250-mm length, 75-μm inside diameter) (packed with 1.8 μm UChrom C18) and separated using a 40-min reverse-phase acetonitrile gradient [5–32% (vol/vol) acetonitrile] with a 250-nL/min flow rate. The mass spectrometer was operated in positive ion mode with a capillary temperature of 220 °C and a 2200 V potential applied to the column. Analysis of +olaparib samples (two TAP-purifications): Peptides were loaded onto an Ultimate 3000 nanoLC system (Dionex/Thermo Scientific); trapped on a precolumn (Acclaim PepMap100, C18, 5 µm, 100 Å, 300 µm i.d. × 5 mm, Thermo Scientific) for 10 min and subsequently separated using an analytical column (Acclaim PepMap100, C18, 5 µm, 100 Å, 75 µm i.d. × 25 cm, Thermo Scientific) employing a 130 min gradient (0–10 min: 5% buffer B (buffer A: 0.1% FA; buffer B: 80% acetonitrile, 0.1% FA), 10–105 min: 10–45% buffer B, 105–107 min: 45–100% buffer B; 107–113 100% buffer B; 113–130 min 5% buffer B). All samples were measured in duplicate on an Orbitrap Elite mass spectrometer (Thermo Scientific). MS settings: full scan spectra (Orbitrap) range: m/z 350 to m/z 1500, with a resolution of 120000 and an AGC setting of 5e5 ions; data dependent mode; 20 s dynamic exclusion; top 10 precursor fragmentation in the ion trap with a collision energy of 35%.

Analysis of the raw data was performed using MaxQuant (version 1.5.1.2,^74^) with the built-in Andromeda search engine^75^. The spectra were searched against the human SwissProt database version 06/2015 (canonical and isoforms). The MaxQuant default settings (including mass tolerance) were used. Specific settings: Trypsin as the protease (two missed cleavages); Carbamidomethylation (Cys) as the fixed modification; Oxidation (Met), Phosphorylation (Ser, Thr, Tyr) and N-terminal protein acetylation as variable modifications: The false discovery rate was 0.01 on both peptide and protein level and the minimum peptide length was set to seven amino acids. Quantification was done using the label free quantitation algorithm from MaxQuant^21,22^.

Tables were created as follows: data was cleared of reversed hits, contaminants and “only identified by site”. Ratios were calculated from the LFQ values from TARG1-TAP vs. empty vector TAP. A student’s t-test was performed with a minimum p-value of 0.01.

### Co-immunoprecipitation (Co-IP)

1 × 10^6^ HEK293 or HEK293T cells were seeded onto 10 cm dishes and transfected the next day with plasmid DNA as indicated (20 µg DNA in total) by calcium phosphate precipitation for 4–6 h. Cells were washed with HEPES buffer (142 mM NaCl, 10 mM HEPES, 6.7 mM KCl, pH 7.3) before cells were incubated in fresh medium for additional 48 h. All following harvesting and IP steps were performed at 4 °C or on ice. The cells were washed once with PBS, were harvested in 500 µl TAP lysis buffer (described above) and lysed for 30 min under permanent agitation. Lysates were cleared by centrifugation at 16.000 × g for 20 min. 30 µl TAP lysate were kept for input analyses and mixed with SDS sample buffer. Per IP sample 20 µl of anti-FLAG M2 beads (suspension volume) were equilibrated in TAP lysis buffer before incubation with the remaining TAP lysate (~500 µl) for 30 min under constant rotation. Centrifugation of the beads was performed at 500 × g for 2 min. The beads were washed three times with 500 µl TAP lysis buffer. The buffer was removed from the beads completely and the beads were resuspended in 40 µl SDS sample buffer. Co-IP and input samples were incubated for 5 min at 95 °C and analyzed by SDS-PAGE and Western blot.

### Purification of recombinant proteins

*E. coli* BL21 (DE3) were transformed with pGEX-4T1 or pDEST17 plasmids encoding for GST-ALY, His-TARG1 proteins or His-CHIKV-nsP3-macro. Bacteria were cultured at 37 °C in LB medium containing 0.4% glucose and protein expression was induced by addition of IPTG (1 mM) when an OD_600_ of 0.5–0.7 was reached. Purification of His-tagged proteins was performed by immobilized metal ion affinity chromatography (IMAC), all harvesting and purification steps were performed at 4 °C or on ice. Bacteria were pelleted and lysed in IMAC lysis buffer (100 mM HEPES, 500 mM NaCl, 10% glycerol, 10 mM imidazole, 0.5 mM TCEP, 1x protease inhibitor cocktail, 100 µg/ml lysozyme, pH 8.0) for 30 min. After sonication and centrifugation, the lysate was incubated with equilibrated TALON metal affinity resin for 1 h. The beads were washed two times with PBS and once with IMAC wash buffer (20 mM HEPES, 500 mM NaCl, 10% glycerol, 10 mM imidazole, 0.5 mM TCEP, pH 7.5) and eluted with IMAC elution buffer (20 mM HEPES, 500 mM NaCl, 10% glycerol, 500 mM imidazole, 0.5 mM TCEP, pH 7.5). Glutathione affinity purification was performed as described previously^76^.

### Thermal shift assay

The thermal shift assay was carried out in a final volume of 25 µl with 1 µg of recombinant His-TARG1 proteins. First, a mastermix containing the assay buffer (100 mM HEPES, pH 7.5 and 150 mM NaCl), SYPRO orange (1:1,000 final dilution) and recombinant His-tagged protein (1 µg per reaction) was prepared. 22.5 µl of the mixture were pipetted into one reaction tube and filled up with 2.5 µl of ligand solution or diluent as control. Each sample was measured in duplicates in a Rotorgene real-time PCR instrument (Corbett) using the 470 nm channel as the source for excitation and the 610 nm channel for detection with a gain of 7. A temperature scan (melt curve) from 25 °C to 95 °C was performed with temperature increasing by 1 °C/min. Data were analyzed according to^25^ using Boltzmann fitting.

### Cell proliferation

For growth curve analysis, cells were seeded at 5 × 10^4^ cells on 6 cm cell culture dishes in non-selective cell culture medium ±100 ng/ml doxycycline. HeLa *OARD1*^−/−^ HA-TARG1 and HA-TARG1-G43E cells were pre-induced with doxycycline (100 ng/ml) to induce transgene expression before seeding. Fresh doxycycline was added every 48–72 h throughout the analysis. Cells were trypsinized every 24 h and cell number was determined using the CASY® cell counter system (OMNI Life Science).

### Live cell imaging

5 × 10^4^ U2OS cells were seeded onto high glass bottom 35 mm µ-dishes (ibidi) and transfected with plasmids encoding for EGFP or EGFP-TARG1 fusion proteins and mCherry-tagged Histone H2B the next day. Treatment with DMSO or 10 µM olaparib was performed for 2 h before imaging. 48 h after transfection, the dishes were directly mounted onto a Zeiss LSM710 confocal laser scanning microscope and kept at 37 °C and 5% CO_2_. Images shown in Fig. 3a were acquired with a LD C-Apochromat 40x/1.1 W Korr M27 objective with a frame size of 512 × 512 px and a 1.5x digital zoom. EGFP fluorescence was excited with the 488 nm line of an Argon laser (25 mW, 2% output) and detected from 493 nm to 586 nm with a main beam splitter filter MBS 488. mCherry fluorescence was excited with a 561 nm DPSS laser (20 mW, 2% output) and detected from 578 nm to 696 nm with a MBS 458/561 filter. Imaging was performed on a 2.2 µm section, corresponding to 2.17 airy units (AU) in the EGFP track and 1.83 AU in the mCherry track. Images shown in Figs 3b,c and 5 were acquired with a C-Apochromat 63x/1.20 W Korr objective with a frame size of 1024 × 1024 px. Settings for excitation and detection were as described above. For each channel the pinhole diameter was set to 1 AU, corresponding to a 0.9 µm section in the EGFP track and a 1.1 µm section in the mCherry track. The pixel dwell time was 6.3 µs (Fig. 3b) or 3.15 µs (Figs 3c and 5). Time series were performed at 20 cycles with 1 min interval using Definite Focus for focus stabilization.

Intensity values were extracted from unprocessed images using the Zen2012 software (Fig. 3a) or ImageJ 1.47 (Fig. 5, Supplementary Fig. S7)^77^. Normalized intensities as depicted in Fig. 5b and d were measured using the ImageJ Time Series Analyzer V3 plugin. Intensities of circular ROIs covering the nucleolar areas and of three circular ROIs placed randomly throughout the nucleoplasmic area were measured for each time point. To account for changes in focus over time, the mean nucleolar EGFP intensity was normalized to the mean nuclear EGFP intensity at each time point for each cell analyzed. Nucleolar EGFP intensities were normalized to the nucleolar EGFP intensity 1 min after treatment.

Only linear adjustments of the histogram range of single channels were performed to increase the image contrast. Adjustments were performed on an entire image and were equally applied to all images belonging to a single experiment.

### Fluorescence loss in photo-bleaching (FLIP)

Cell seeding and treatments were performed as described for live cell imaging. FLIP experiments were performed with a Zeiss LSM710 confocal laser scanning microscope using a C-Apochromat 63x/1.2 W Korr objective. The 488 nm line of an Argon laser (25 mW, 1% output for imaging scans) was used for excitation of EGFP. EGFP fluorescence was detected from 493 nm to 574 nm. mCherry fluorescence was acquired to localize nuclei and nucleoli. mCherry was excited with a 561 nm DPSS laser (20 mW, 1% output for imaging scans) and fluorescence detected from 578 nm to 696 nm. A MBS 488/561 filter was applied for detection of EGFP and mCherry fluorescence. Imaging and bleaching were performed on a 2 µm section, corresponding to 2.39 AU in the EGFP track and 2 AU in the mCherry track. The frame size was set to 512 × 512 px. The pixel dwell time was 1.27 µs during imaging. Initial photo-bleaching of a circular ROI (∅2.8 µm, width and height of 11 px) was performed after 5 imaging scans using the 488 nm laser at 100% intensity with 100 iterations applying a different scan speed (pixel dwell time of 25.21 µs) and the zoom bleach option. Bleaching was repeated after every imaging scan. Nuclear photo-bleaching was performed for 60 s, after which imaging was continued without photo-bleaching for additional 30 s. The average fluorescence intensities in each of the defined ROIs were measured during imaging using the Zen2012 software. The ROI area was kept constant for every sample and cells of similar size were chosen. At least 7 data sets were analyzed per sample. Fluorescence intensities in each ROI were normalized to the mean fluorescence intensity in the same ROI before bleaching.

### RNA binding assay

RNA EMSAs were performed as described previously^41^. Briefly, pcDNA3.1-based Pentaprobe plasmids were linearized by *Apa*I followed by a Klenow-fill in of 3′ overhangs. 1 µg of purified, linearized plasmid DNA was *in vitro* transcribed using the T7 RiboMAX™ Large Scale RNA Production System (Promega) in the presence of α-^32^P-UTP. The *in vitro* transcription reaction was separated on denaturing urea-TBE polyacrylamide gels. The gel slice containing the labeled RNA Pentaprobe fragment was crushed and soaked in H_2_O over night. Samples were centrifuged and the supernatant containing the labeled RNA was transferred to a new reaction tube. The soaking step was repeated for 2 h. Supernatants from both soaking steps were combined and the RNA was ethanol-precipitated over night at −20 °C. The precipitation reaction was centrifuged for 30 min at 4 °C at 13,000 × g. The ethanol was removed carefully and the RNA pellet incubated at 37 °C until residual ethanol was evaporated completely. The RNA pellet was resuspended in 100–150 µl TBE buffer and incubated at 37 °C for 5–10 min. Probes were stored at −20 °C until use and, directly before use, were heated for 45 s at 95 °C and put straight on ice. RNA binding reactions were carried out in a 30 µl volume with 1 µl of labeled RNA and 0–20 pmol of recombinant protein in gel shift buffer (10 mM MOPS pH 7.0, 50 mM KCl, 5 mM MgCl_2_, 1 mM DTT, 10% glycerol) for 30 min at 4 °C. For antibody supershift experiments, 500 ng of an anti-TARG1 antibody (polyclonal, Eurogentec) were added prior to addition of the labeled RNA. The binding reactions were loaded onto 7% acrylamide/bisacrylamide (19:1) gels and electrophoresed in TB buffer (45 mM Tris, 45 mM boric acid) at 10 mA at 4 °C for 2–3 h. Gels were dried and analyzed by auto-radiography.

### Preparation of whole cell lysates for western blot analyses

For the preparation of whole cell lysates, cells were harvested in RIPA buffer (10 mM Tris, pH 7.4, 150 mM NaCl, 1% (v/v) Nonidet P-40, 1% (v/v) deoxycholate, 0.1% SDS, 1x protease inhibitor cocktail) and sonicated. Cell lysates were centrifuged at 16,000 × g, 4 °C for 20 min. The supernatant was transferred to a fresh reaction tube, mixed with SDS sample buffer, heated for 5 min at 95 °C and analyzed by SDS-PAGE/Western blotting.

### Data availability

The mass spectrometry proteomics data (raw MS data), MaxQuant output txt files and the corresponding fasta file have been deposited to the ProteomeXchange Consortium (http://proteomecentral.proteomexchange.org) via the PRIDE partner repository^78^ with the dataset identifier PXD008748.

## Electronic supplementary material

Supplementary Information

Dataset S1

Dataset S2
