# Supplementary material for: Nucleolar-nucleoplasmic shuttling of TARG1 and its control by DNA damage-induced poly-ADP-ribosylation and by nucleolar transcription
Source: Sci Rep. 2018 Apr 30;8:6748. doi: 10.1038/s41598-018-25137-w (PMC5928194; doi:10.1038/s41598-018-25137-w)
Supplement: Supplementary file 1 — Supplementary Information [file 41598_2018_25137_MOESM1_ESM.pdf]

Supplementary Information

Nucleolar-nucleoplasmic shuttling of TARG1 and its control by DNA damage-induced poly-ADP-ribosylation and by nucleolar transcription

Mareike Bütepage[1], Christian Preisinger[2], Alexander von Kriegsheim[3,9], Anja Scheufen[1], Eva Lausberg[1,9], Jinyu Li[1,9], Ferdinand Kappes[1,9], Regina Feederle[4], Sabrina Ernst[1,5], Laura Eckei[1], Sarah Krieg[1], Gerhard Müller-Newen[1,5], Giulia Rossetti[6,7,8], Karla Feijs[1], Patricia Verheugd[1] and Bernhard Lüscher[1,*]

[1] Institute of Biochemistry and Molecular Biology, Medical School, RWTH Aachen University, Pauwelsstraße 30, 52074 Aachen, Germany

[2] Proteomics Facility, Interdisciplinary Centre for Clinical Research (IZKF), Medical School, RWTH Aachen University, Pauwelsstraße 30, 52074 Aachen, Germany

[3] Systems Biology Ireland, Conway Institute, University College Dublin, Dublin 4, Ireland

[4] Institute for Diabetes and Obesity, Monoclonal Antibody Core Facility, Helmholtz Center Munich, German Research Center for Environmental Health, Ingolstädter Landstrasse 1, Neuherberg, Germany

[5] Immunohistochemistry and Confocal Microscopy Facility, Interdisciplinary Centre for Clinical Research (IZKF), Medical School, RWTH Aachen University, Pauwelsstraße 30, 52074 Aachen, Germany

[6] Computational Biomedicine, Institute for Advanced Simulation IAS-5 and Institute of Neuroscience and Medicine INM-9, Forschungszentrum Jülich, 52425 Jülich, Germany

[7] Jülich Supercomputing Centre, Forschungszentrum Jülich, 52425 Jülich, Germany

[8] Department of Oncology, Hematology and Stem Cell Transplantation, Medical School, RWTH Aachen University, Pauwelsstraße 30, 52074 Aachen, Germany

[9] current addresses: Edinburgh Cancer Research Centre, IGMM, University of Edinburgh, Edinburgh EH4 2XR, UK (AvK); Institute of Human Genetics, Medical School, RWTH Aachen University, Pauwelsstraße 30, 52074 Aachen, Germany (EL); College of Chemistry, Fuzhou University, 350116 Fuzhou, China (JL); Department of Biological Sciences, Xi'an Jiaotong-Liverpool University, No 111, Ren Ai Road, Dushu Lake Higher Education Town, Suzhou Industrial Park, Suzhou, 215123, P.R. China (FK)

* Correspondence: luescher@rwth-aachen.de; Tel.: +49-241-8088850; Fax: +49-241-8082427; ORCID: 0000-0002-9622-8709.

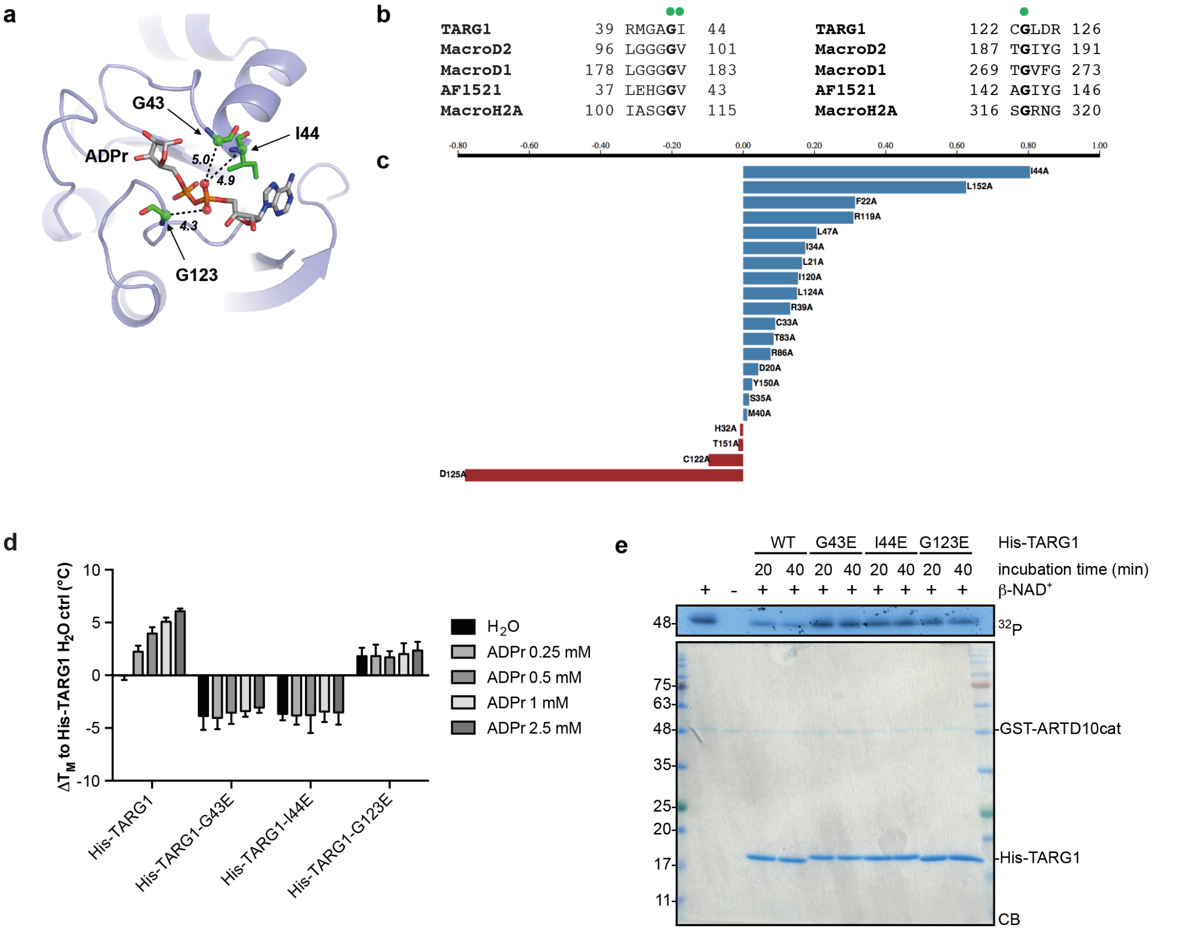

Supplementary Figure S1: Establishment of ADPr binding-deficient TARG1 mutants

(a) NMR structure of TARG1/ADPr (PDB ID: 2L8R [1]). TARG1 and ADPr are shown in blue cartoon and gray stick models, respectively. The mutagenesis sites tested experimentally (G43, I44 and G123) are highlighted in green stick model. The closest distances (in Å) between the Cα atoms of these residues and the oxygen atoms of ADPr phosphates are indicated by black dashed lines.

(b) Sequence alignments of macrodomains around the mutagenesis sites (G43, I44 and G123 of TARG1, indicated by green solid circles).

(c) *In silico* alanine scan and ΔΔG values for ADPr binding.

(d) Thermal stability of TARG1 and mutants in the presence of ADPr. 2 µM His-TARG1, His-TARG1-G43E, -I44E or -G123E were incubated with increasing amounts of ADPr (0.25 mM – 2.5 mM) and the fluorescent dye SYPRO orange. SYPRO orange fluorescence intensity was measured in a qPCR light cycler with a temperature gradient from 25°C to 95°C with temperature increments of 1°C/min. Melting temperatures were determined according to [2] and are presented as ΔT_M (mean + SD of 3 experiments) to His-TARG1 H_2O control (ctrl).

(e) Hydrolase activity of His-TARG1 and mutants. Immobilized GST-coupled ARTD10 catalytic domain was auto-modified in the presence of ^{32}P-β-NAD$^+$ and incubated with His-TARG1 (WT) or His-TARG1-G43E, -I44E or -G123E for 20 or 40 min. Proteins were separated by SDS-PAGE. Removal of the incorporated radioactive label of ARTD10cat was monitored by auto-radiography (^{32}P). CB: Coomassie blue.

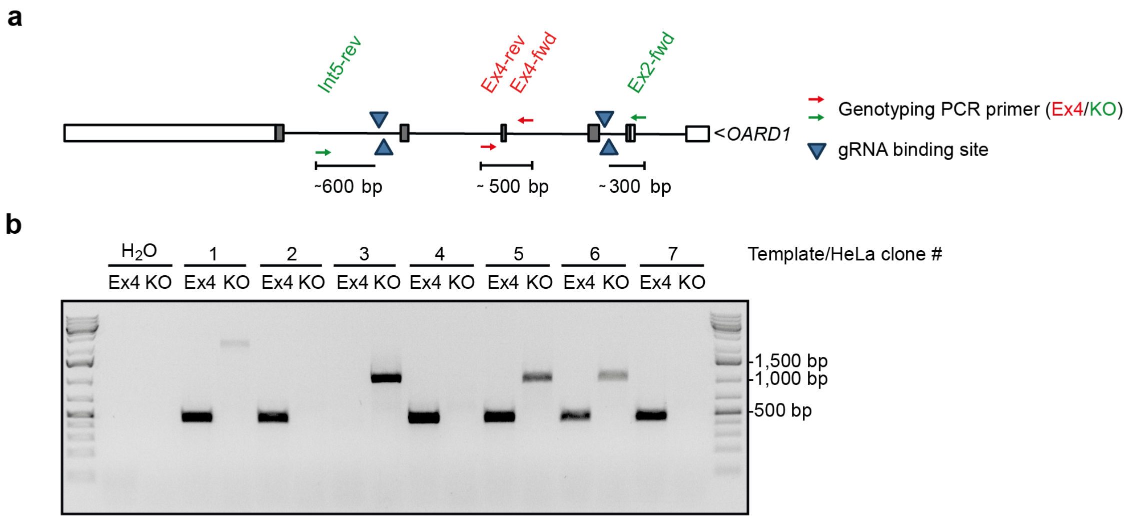

Supplementary Figure S2: CRISPR-Cas9 *OARD1* knock-out strategy

(a) Schematic representation of the *OARD1* locus on the reverse strand of chromosome 6 and knock-out strategy. HeLa cells were transfected with plasmids encoding for the Cas9 nickase (Cas9-D10A) and gRNAs targeting Cas9-D10A to the forward and the reverse strand in intron 2 and intron 5 each of the *OARD1* gene (blue triangles) resulting in deletion of exons 3-5. Genotyping of single cell clones was performed by genomic PCR. The wildtype allele was detected using the primer pair amplifying exon 4 (Ex4, red) while upon deletion of the gDNA-flanked fragment a PCR product of ~1,000 bp can be amplified using the primer pair Ex2-fwd/Int5-rev (KO, green).

(b) Exemplary agarose gel of genotyping PCR using genomic DNA samples from 7 different HeLa cell clones as a template and the primer pairs described in (A) for amplification of the wildtype (Ex4) and the knock-out (KO) allele. A complete knock-out was observed for clone 3.

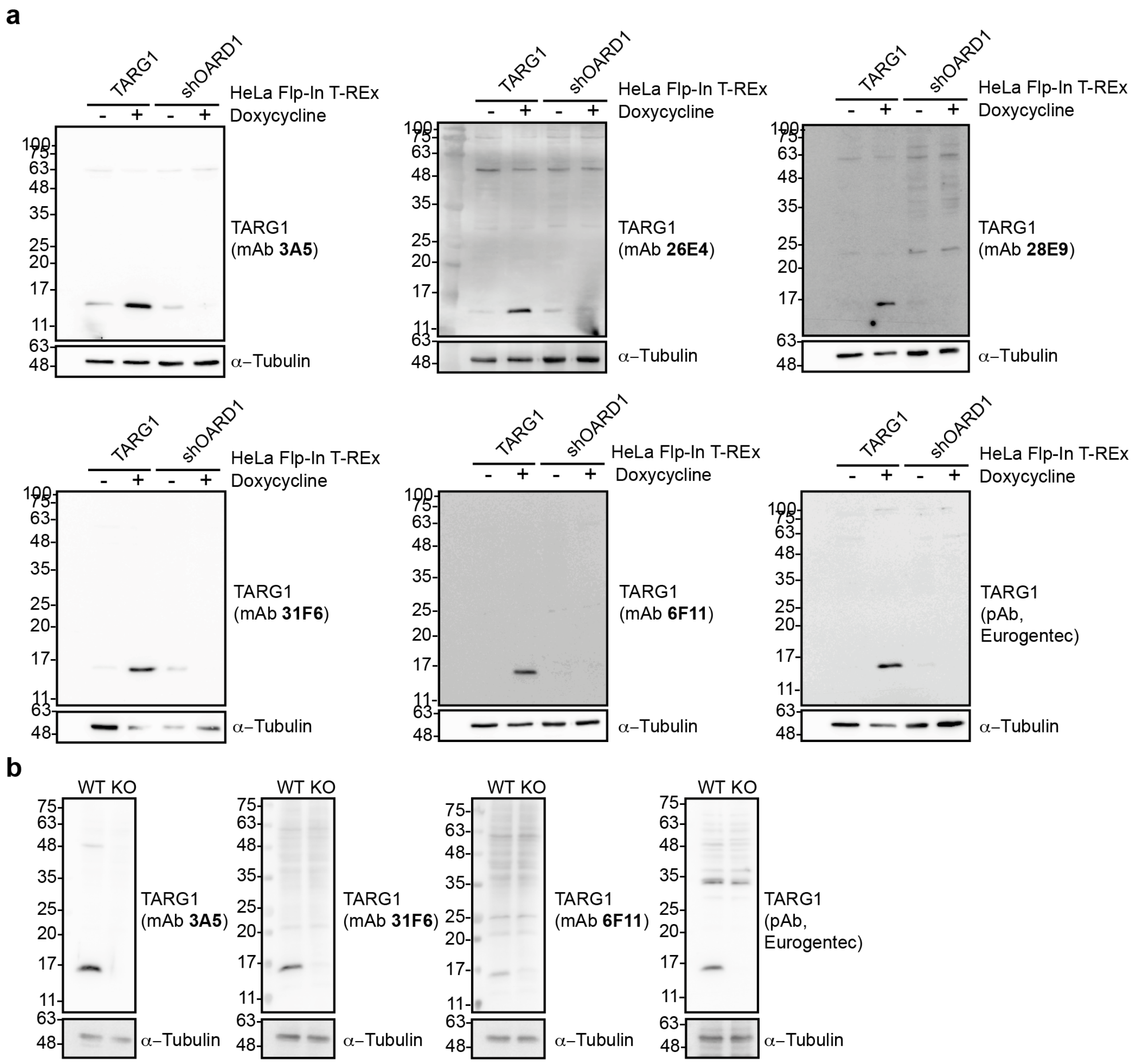

Supplementary Figure S3: Validation of TARG1 antibodies

(a) TARG1 protein expression in whole cell lysates of HeLa Flp-In™ T-REx™ cells inducibly expressing either TARG1 or an shRNA against *OARD1* mRNA (shOARD1) upon doxycycline addition was analyzed by SDS-PAGE/Western blot with monoclonal antibodies generated against full-length TARG1 (3A5, rat; 26E4, mouse; 28E9, mouse; 31F6, mouse; 6F11, rat) or a polyclonal antibody raised against two peptides in the C-terminus of TARG1 (rabbit, Eurogentec).

(b) TARG1 protein expression in whole cell lysates of HeLa (WT) and HeLa *OARD1*$^{-/-}$ (KO; cl. 3) cells was analyzed by SDS-PAGE/Western blot with the antibodies described in (a).

(a and b) α-Tubulin levels were detected as a loading control. Full-length blots are presented in Supplementary Fig. S9.

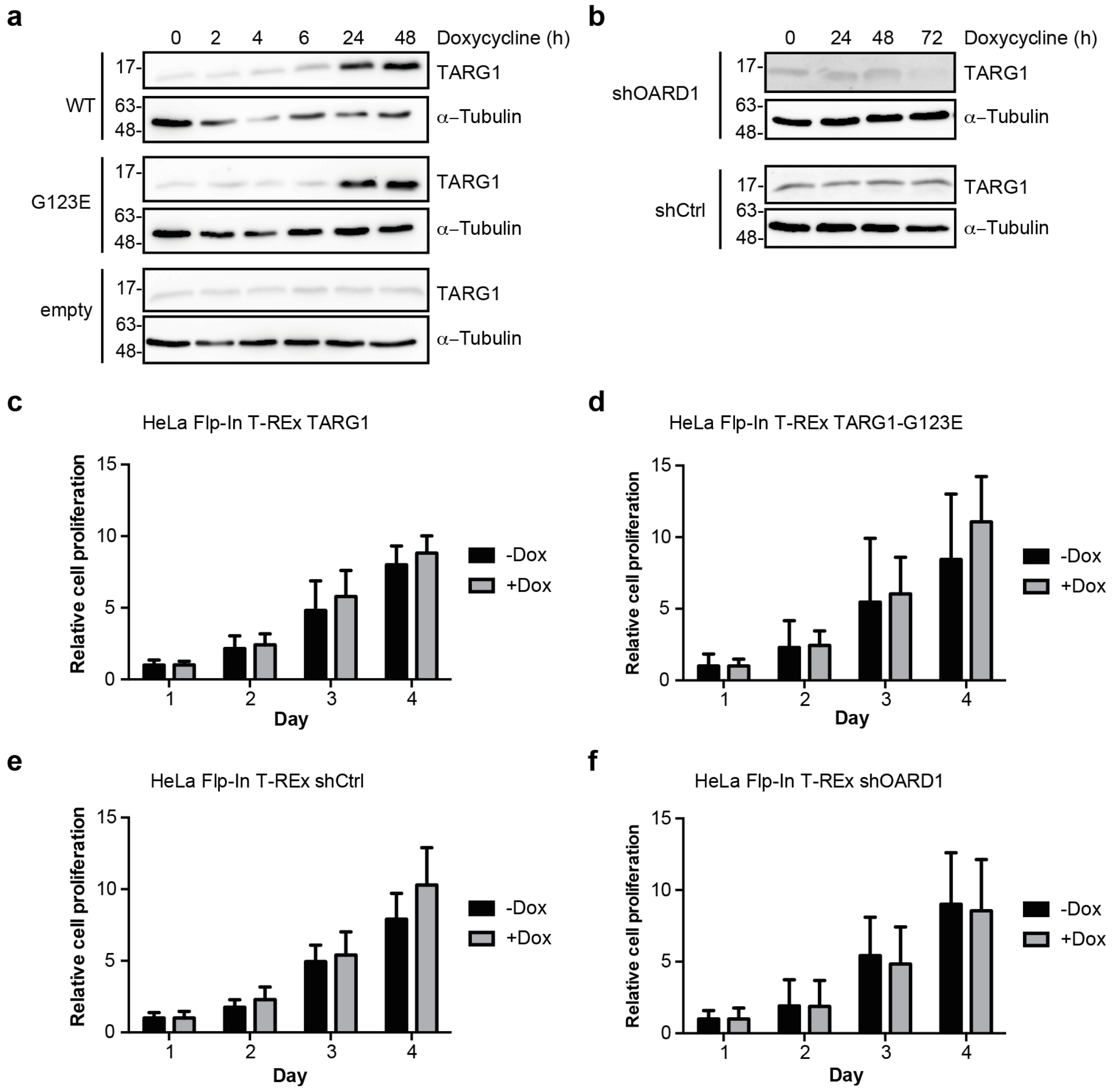

Supplementary Figure S4: Cell proliferation analyses of HeLa cells with inducible overexpression or knock-down of TARG1

(a) HeLa Flp-In™ T-REx™ cell lines stably expressing TARG1 wildtype (WT), TARG1-G123E (G123E) or an empty vector control were treated with 500 ng/ml doxycycline for the indicated time points to induce transgene expression. Whole cell lysates were prepared and TARG1 protein expression analyzed by Western blotting using a polyclonal TARG1 antibody (Eurogentec). Exogenous TARG1 WT and G123E are detectable 24 h after doxycycline treatment while no increase in TARG1 expression is observed in the empty vector control. α-Tubulin levels were detected as a loading control.

(b) HeLa Flp-In™ T-REx™ cell lines stably expressing an shRNA against *OARD1* mRNA (shOARD1) or a control shRNA (shCtrl) were treated with 500 ng/ml doxycycline for the indicated time points to induce shRNA expression. Whole cell lysates were prepared and TARG1 protein expression analyzed by Western blotting using a polyclonal TARG1 antibody (Eurogentec). Reduction in TARG1 expression was detected in HeLa cells expressing shOARD1 72 h after doxycycline induction but not in shCtrl expressing HeLa cells. α-Tubulin levels were detected as a loading control.

(c-f) HeLa Flp-In™ T-REx™ cells inducibly expressing TARG1 (c), -G123E (d), shOARD1 (e) or shCtrl (f) were or were not treated with 500 ng/ml doxycycline (Dox) for 72 h to induce transgene expression or *OARD1* knock-down before seeding onto 96 well-plates. Cell viability was measured 1, 2, 3 and 4 days after seeding using the cell proliferation reagent WST-1 (mean ± SD of 3 experiments).
(a and b) Full-length blots are presented in Supplementary Fig. S9.

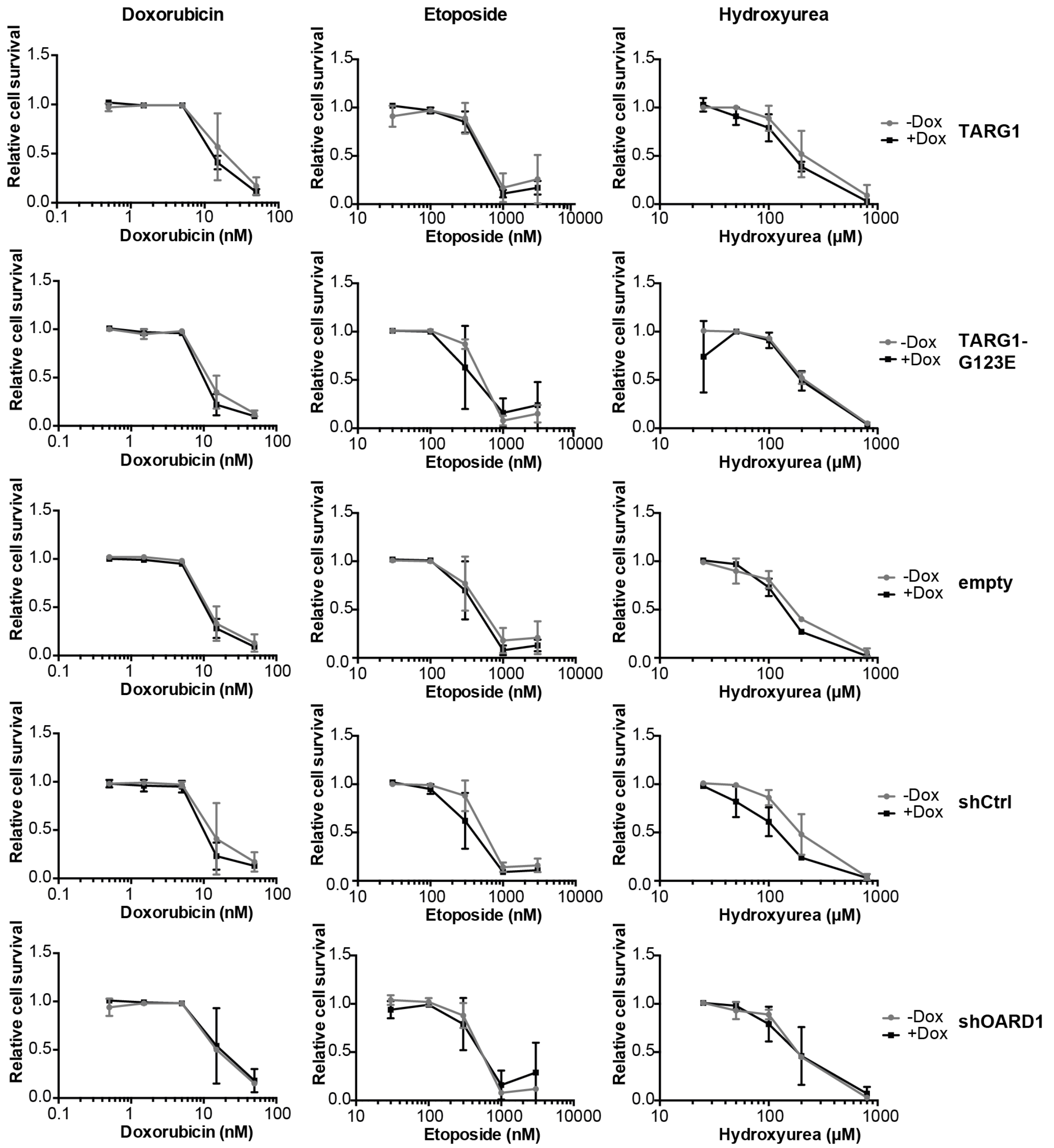

Supplementary Figure S5: Sensitivity of HeLa cells with inducible overexpression or knock-down of TARG1 to DNA damaging agents

HeLa Flp-In™ T-REx™ cells inducibly expressing TARG1, TARG1-G123E, an empty vector control (empty), shCtrl or shOARD1 were or were not treated with 500 ng/ml doxycycline (-/+Dox) for 72 h to induce transgene expression or *OARD1* knock-down before seeding the cells in 6-well plates in cell culture medium -/+ doxycycline. 24 h after seeding, the cells were treated with doxorubicine for 24 h, or etoposide or hydroxyurea for 48 h at the indicated concentrations. Relative cell survival on day 4 after seeding was calculated as described in the supplementary methods section (mean ± SD of 2 biological replicates measured in duplicates).

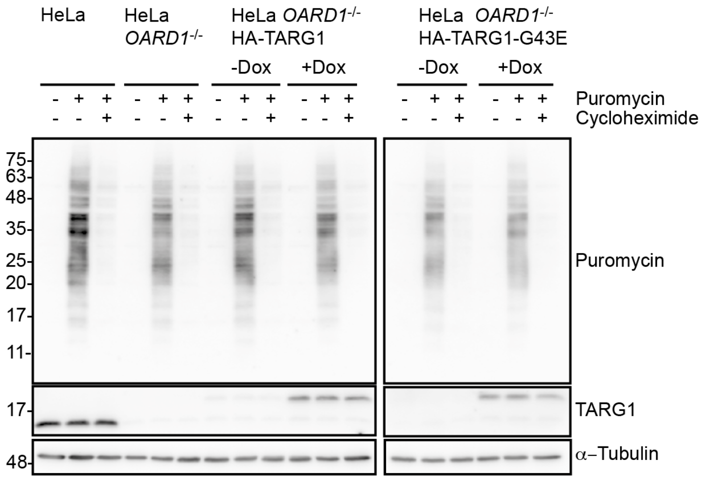

Supplementary Figure S6: Translation activity of HeLa OARD1 knock-out cells.
Translation activity of HeLa *OARD1* knock-out cells was estimated by a puromycin incorporation assay [3]. HeLa, HeLa *OARD1*$^{-/-}$, HeLa *OARD1*$^{-/-}$ HA-TARG1, HeLa *OARD1*$^{-/-}$ HA-TARG1-G43E were or were not induced by treatment with 100 ng/ml doxycycline as indicated. 48 h after doxycycline induction, the cells were treated with 10 µg/ml puromycin for 10 min to allow puromycin incorporation into nascent polypeptides. The cells were incubated for further 50 min at 37°C without puromycin and harvested. Puromycin incorporation was analyzed in whole cell lysates by SDS-PAGE/Western blot using a monoclonal puromycin antibody (12D10). Pre-treatment of the cells with 25 µg/ml cycloheximide blocked puromycin incorporation into nascent polypeptides. TARG1 protein expression was analyzed with the monoclonal TARG1 antibody 3A5. α-Tubulin was analyzed as a loading control. Full-length blots are presented in Supplementary Fig. S9.

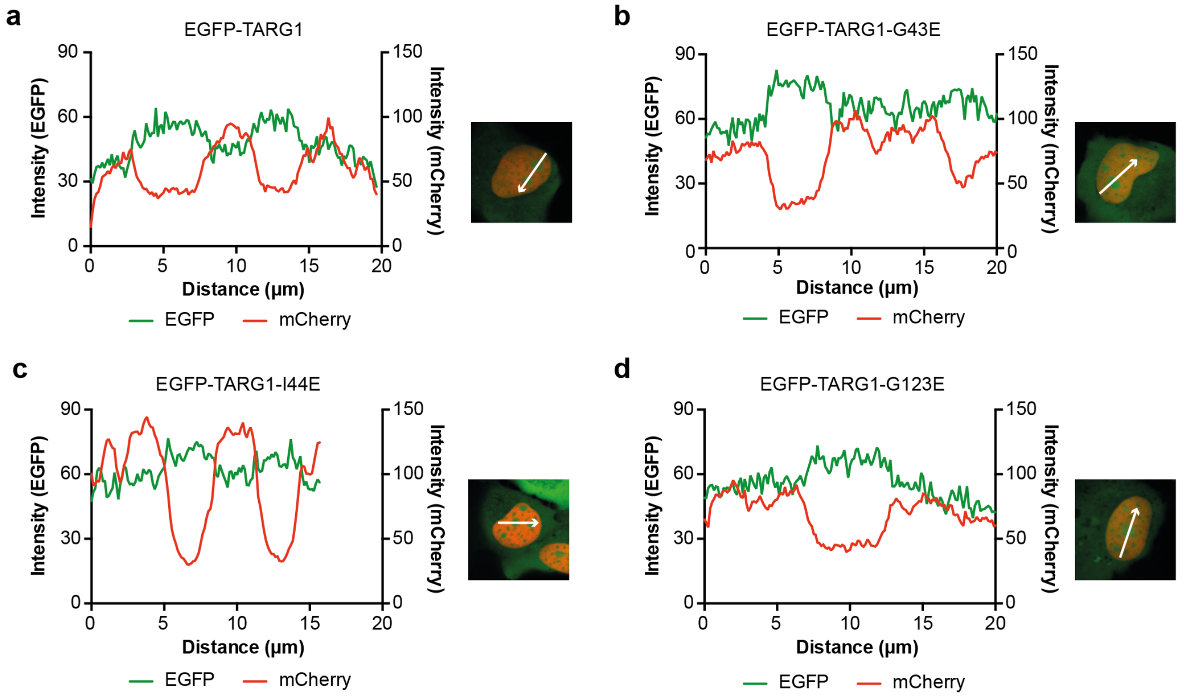

Supplementary Figure S7

(a-d) Intensity profiles for EGFP and mCherry fluorescence signals of the images presented in Figure 3b along the depicted arrows.

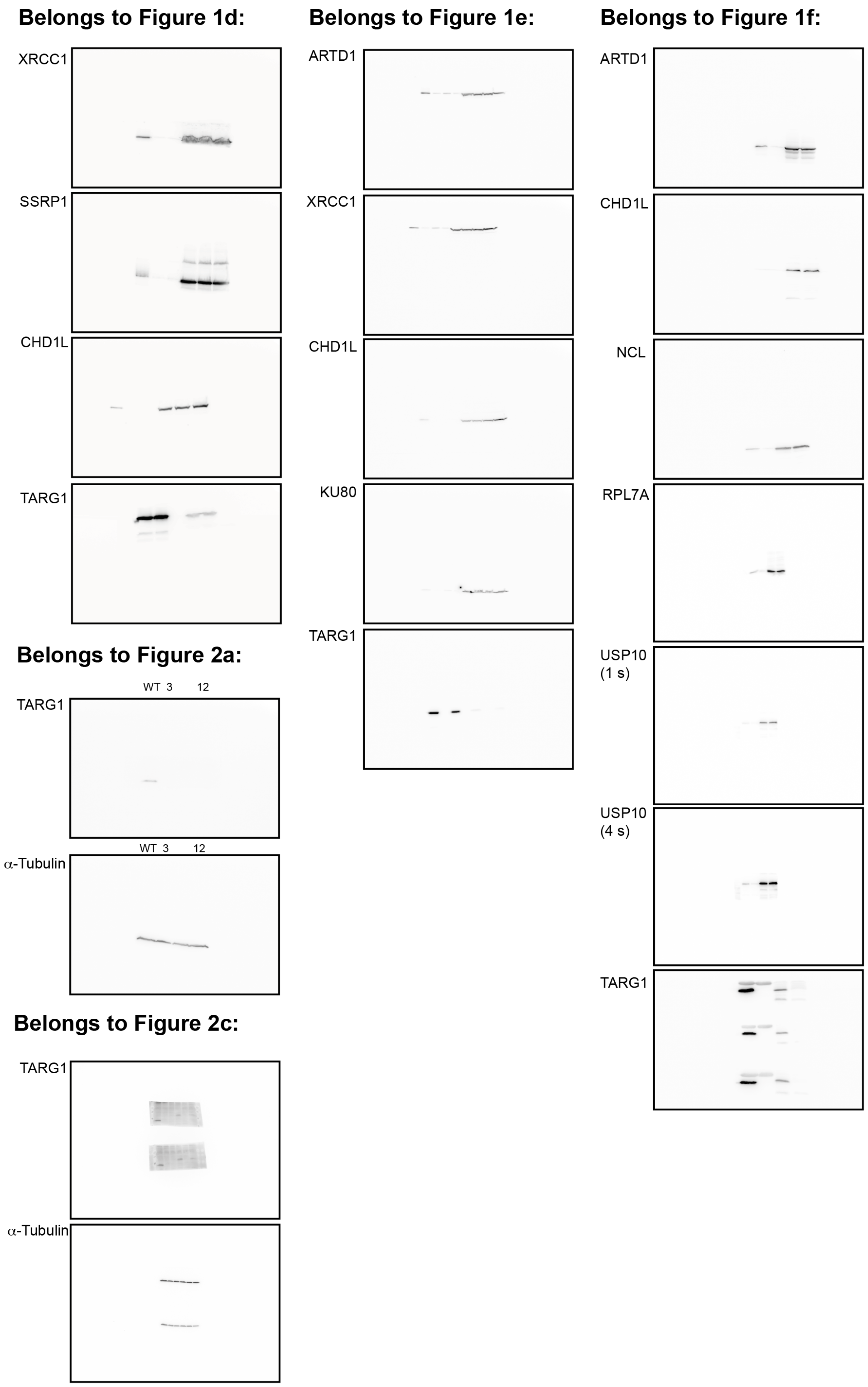

Supplementary Figure S8

Uncropped original images of Western blots are shown belonging to Figures 1d-f, 2a and 2c.

Belongs to Figure S3a:

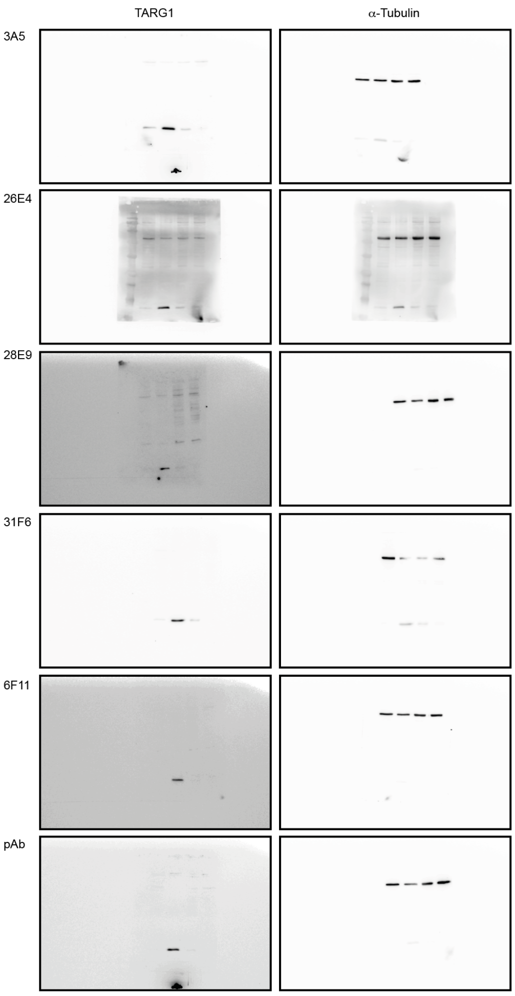

Belongs to Figure S3b:

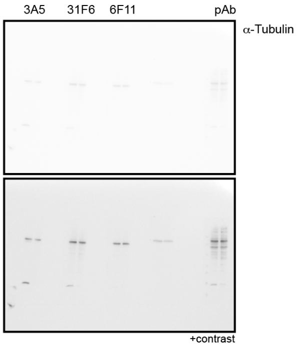

Belongs to Figure S4a:

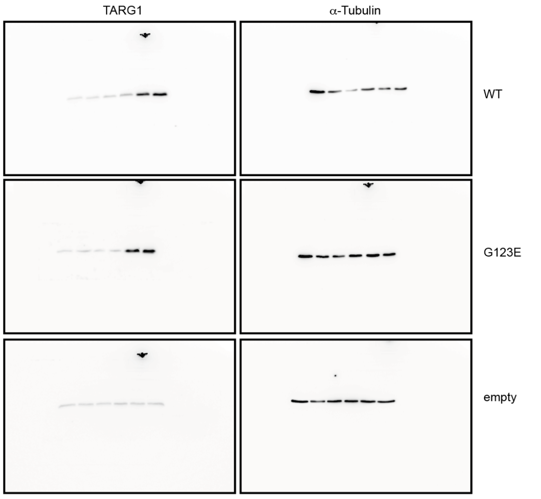

Belongs to Figure S4b:

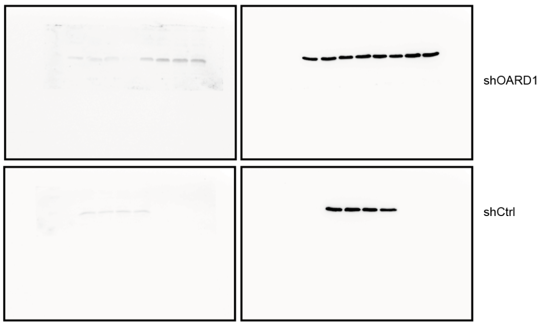

Belongs to Figure S6:

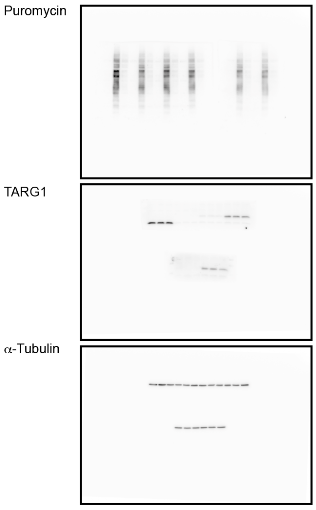

Supplementary Figure S9

Uncropped original images of Western blots are shown belonging to Figures S3, S4 and S6.

Supplementary Table S1

Proteins enriched in the TAP-pulldown experiments +/- olaparib (minimum two-fold). The raw mass spectrometry data was analysed using MaxQuant as described in materials and methods. The output file "proteinGroups.txt" was used for further analysis. The ratio was calculated as the mean of all LFQ intensities of TAP-TARG/empty TAP-vector. If no LFQ intensity was reported the levels of "0" were set to "1" in order to avoid DIV/0 errors when calculating the ratio.

TAP-Purification of TARG/OARD1 minus Olaparib

Majority protein IDs	Protein names	Gene names	Razor + unique peptides	Unique peptides	log_2 LFQ ratio	ttest
Q01831	DNA repair protein complementing XP-C cells	XPC	7	7	31.74	3.80E-04
Q7Z7B0-3	Filamin-A-interacting protein 1	FILIP1	1	1	29.83	5.42E-03
O75531	Barrier-to-autointegration factor	BANF1	5	5	28.59	1.63E-03
F5H8D7	DNA repair protein XRCC1	XRCC1	8	8	28.23	1.34E-06
Q86WJ1-2	Chromodomain-helicase-DNA-binding protein 1-like	CHD1L	15	1	28.19	3.29E-05
Q14191	Werner syndrome ATP-dependent helicase	WRN	14	14	28.03	7.75E-09
Q8NC56	LEM domain-containing protein 2	LEMD2	6	6	26.81	2.00E-03
Q5TEC6	Histone H3.3C	HIST2H3PS2	1	1	26.77	1.21E-03
P62979	Ubiquitin-40S ribosomal protein S27a	RPS27A	3	3	26.48	2.49E-03
Q13472-2	DNA topoisomerase 3-alpha	TOP3A	4	4	26.29	7.00E-05
P42166	Lamina-associated polypeptide 2, isoform alpha	TMPO	2	2	24.94	1.32E-04
Q9Y530	O-acetyl-ADP-ribose deacetylase 1	OARD1	28	4	16.68	1.44E-03
P12956	X-ray repair cross-complementing protein 6	XRCC6	44	44	11.86	2.09E-04
P09874	Poly [ADP-ribose] polymerase 1	PARP1	86	86	8.59	2.61E-04
A0A0C4DGA6	Helicase-like transcription factor	HLTF	28	28	8.51	2.22E-03
P13010	X-ray repair cross-complementing protein 5	XRCC5	38	38	7.08	1.61E-04
P49916-3	DNA ligase 3	LIG3	27	27	6.13	8.23E-05
P11388	DNA topoisomerase 2-alpha	TOP2A	5	5	5.66	5.68E-03
P35244	Replication protein A 14 kDa subunit	RPA3	4	4	5.27	9.17E-03
Q08945	FACT complex subunit SSRP1	SSRP1	44	44	5.12	1.43E-04
Q9Y5B9	FACT complex subunit SPT16	SUPT16H	79	79	5.09	6.31E-08
Q93077	Histone H2A type 1-C	HIST1H2AC	2	2	4.85	9.89E-03
Q9BUH6	Uncharacterized protein C9orf142	C9orf142	7	7	4.76	2.16E-03
P05412	Transcription factor AP-1	JUN	5	4	4.74	8.26E-04
Q8N4S0	Coiled-coil domain-containing protein 82	CCDC82	6	6	4.53	1.25E-04
Q01105-3	Protein SET	SET	2	2	4.41	4.64E-08
P17535	Transcription factor jun-D	JUND	2	2	4.18	8.04E-03
P39880-3	Homeobox protein cut-like 1	CUX1	33	33	4.14	6.18E-05
P35250	Replication factor C subunit 2	RFC2	3	3	4.13	1.15E-07
P62805	Histone H4	HIST1H4A	12	12	3.88	1.43E-03
J3QSH4	Vascular endothelial zinc finger 1	VEZF1	4	3	3.84	3.65E-04
K7EK07	Histone H3	H3F3B	6	2	3.82	3.06E-04
Q99878	Histone H2A type 1-J	HIST1H2AJ	6	2	3.70	6.40E-04
Q6SPF0	Atherin	SAMD1	13	13	3.66	4.34E-05
P78527	DNA-dependent protein kinase catalytic subunit	PRKDC	135	135	3.32	2.82E-04
Q71UI9	Histone H2A.V	H2AFV	3	3	3.29	9.53E-03
Q99879	Histone H2B type 1-M	HIST1H2BM	10	2	2.92	3.33E-04
P35251-2	Replication factor C subunit 1	RFC1	5	5	2.79	1.35E-04
Q99627-2	COP9 signalosome complex subunit 8	COPS8	2	2	2.67	9.02E-03
P50402	Emerin	EMD	10	10	2.56	2.06E-03
P40937-2	Replication factor C subunit 5	RFC5	3	3	2.53	6.08E-04
P40938-2	Replication factor C subunit 3	RFC3	3	3	2.34	6.08E-09
Q92522	Histone H1x	H1FX	6	6	2.31	2.26E-03
Q00059	Transcription factor A, mitochondrial	TFAM	17	17	2.24	2.42E-05
P47914	60S ribosomal protein L29	RPL29	4	4	2.08	8.31E-04
P16403	Histone H1.2	HIST1H1C	13	3	2.08	6.14E-06
P42167	Lamina-associated polypeptide 2, isoforms beta/gamma	TMPO	10	4	1.81	4.33E-04
Q8WXI9	Transcriptional repressor p66-beta	GATAD2B	5	4	1.79	1.43E-03
P46777	60S ribosomal protein L5	RPL5	13	13	1.72	1.78E-03
C9JTT7	Replication factor C subunit 4	RFC4	4	4	1.67	1.01E-04
P39023	60S ribosomal protein L3	RPL3	19	19	1.66	2.62E-03
P62424	60S ribosomal protein L7a	RPL7A	12	12	1.62	6.32E-04
Q07020-2	60S ribosomal protein L18	RPL18	6	6	1.62	1.78E-03
P61313	60S ribosomal protein L15	RPL15	10	10	1.60	2.46E-04
E9PBF6	Lamin-B1	LMNB1	7	7	1.57	3.03E-03
P30050	60S ribosomal protein L12	RPL12	6	6	1.56	3.43E-03
Q02878	60S ribosomal protein L6	RPL6	10	10	1.55	1.44E-03
P18124	60S ribosomal protein L7	RPL7	10	10	1.52	1.21E-03
F8VW21	60S acidic ribosomal protein P0	RPLP0	6	6	1.51	6.38E-04

P62917	60S ribosomal protein L8	RPL8	11	11	1.51	4.29E-03
P49207	60S ribosomal protein L34	RPL34	5	5	1.50	7.71E-03
P19338	Nucleolin	NCL	25	25	1.49	6.09E-04
J3QR09	Ribosomal protein L19	RPL19	9	9	1.42	1.08E-03
M0R3D6	60S ribosomal protein L18a	RPL18A	5	5	1.41	9.55E-03
P36578	60S ribosomal protein L4	RPL4	16	16	1.38	4.87E-03
P62750	60S ribosomal protein L23a	RPL23A	11	11	1.34	7.29E-05
Q9Y3U8	60S ribosomal protein L36	RPL36	4	4	1.33	4.55E-03
D6RAN4	60S ribosomal protein L9	RPL9	9	9	1.27	9.32E-04
P05387	60S acidic ribosomal protein P2	RPLP2	4	3	1.26	3.21E-04
P67809	Nuclease-sensitive element-binding protein 1	YBX1	8	6	1.16	2.13E-03
P06748-2	Nucleophosmin	NPM1	9	9	1.05	9.50E-03

TAP-Purification of TARG/OARD1 plus Olaparib

Majority protein IDs	Protein names	Gene names	Razor + unique peptides	Unique peptides	$\log_2$ LFQ ratio	ttest
H7C2W9	60S ribosomal protein L31	RPL31	3	3	29.75	7.81E-06
P05386	60S acidic ribosomal protein P1	RPLP1	2	2	28.31	8.34E-04
Q96P11	Putative methyltransferase NSUN5	NSUN5	10	10	28.18	2.49E-04
Q9NR30-2	Nucleolar RNA helicase 2	DDX21	11	10	28.18	6.39E-07
Q7L2E3-3	Putative ATP-dependent RNA helicase DHX30	DHX30	11	11	27.62	7.44E-09
P98179	Putative RNA-binding protein 3	RBM3	3	3	27.29	2.36E-04
Q8IY37	Probable ATP-dependent RNA helicase DHX37	DHX37	7	7	27.28	2.42E-04
Q9BQ67	Glutamate-rich WD repeat-containing protein 1	GRWD1	4	4	27.22	1.77E-04
A0A0B4J220			3	3	26.90	8.95E-05
E5RJU9	Protein LYRIC	MTDH	5	5	26.88	9.09E-03
F8WBS5	60S ribosomal protein L35a	RPL35A	2	2	26.72	1.10E-05
Q9NUL7	Probable ATP-dependent RNA helicase DDX28	DDX28	4	4	26.63	1.13E-04
Q9H501	ESF1 homolog	ESF1	5	5	26.61	1.91E-04
P11387	DNA topoisomerase 1	TOP1	5	5	26.61	5.51E-07
Q99755-2	Phosphatidylinositol 4-phosphate 5-kinase type-1 alpha	PIP5K1A	3	3	26.48	7.26E-07
O14979-3	Heterogeneous nuclear ribonucleoprotein D-like	HNRPDL	3	3	26.44	2.46E-03
Q8TDN6	Ribosome biogenesis protein BRX1 homolog	BRIX1	3	3	26.36	2.10E-03
Q9H6F5	Coiled-coil domain-containing protein 86	CCDC86	2	2	26.10	2.01E-04
Q9NVN8	Guanine nucleotide-binding protein-like 3-like protein	GNL3L	4	4	25.92	3.39E-06
Q9UL40	Zinc finger protein 346	ZNF346	3	3	25.83	5.69E-09
Q96GA3	Protein LTV1 homolog	LTV1	3	3	25.62	1.32E-05
R9WNI0	Fragile X mental retardation protein 1	FMR1	3	3	25.57	6.22E-03
Q8WU90	Zinc finger CCCH domain-containing protein 15	ZC3H15	3	3	25.52	1.89E-06
A0A087X1Q1	Cellular tumor antigen p53	TP53	2	2	25.34	7.66E-04
S4R3G0	Serine/arginine-rich splicing factor 9	SRSF9	2	2	25.31	7.36E-07
F8WDT8	Probable ATP-dependent RNA helicase DDX56	DDX56	2	2	25.28	5.63E-07
Q9Y2Z2-2	Protein MTO1 homolog, mitochondrial	MTO1	4	4	24.89	8.77E-04
Q5JR04	Putative helicase MOV-10	MOV10	3	3	24.88	6.93E-05
H7C446	Suppressor of SWI4 1 homolog	PPAN	2	2	24.87	5.66E-06
H0YFD2	Protein KRI1 homolog	KRI1	2	2	24.68	9.72E-06
Q49A26-5	Putative oxidoreductase GLYR1	GLYR1	1	1	23.38	1.56E-06
Q9Y530	O-acetyl-ADP-ribose deacetylase 1	OARD1	25	3	12.37	4.24E-10
P39023	60S ribosomal protein L3	RPL3	13	13	4.90	1.03E-03
P06748-2	Nucleophosmin	NPM1	9	9	4.41	1.23E-03
P46779-4	60S ribosomal protein L28	RPL28	4	4	4.18	2.98E-05
Q59GN2	Putative 60S ribosomal protein L39-like 5	RPL39P5	2	2	4.07	2.06E-04
P61353	60S ribosomal protein L27	RPL27	7	7	4.06	8.71E-04
E7EPB3	60S ribosomal protein L14	RPL14	2	2	4.05	2.80E-04
Q02878	60S ribosomal protein L6	RPL6	15	15	4.03	3.87E-04
P36578	60S ribosomal protein L4	RPL4	15	15	3.90	7.82E-04
P46777	60S ribosomal protein L5	RPL5	12	12	3.88	2.72E-05
M0R3D6	60S ribosomal protein L18a	RPL18A	5	5	3.87	1.28E-04
Q96AG4	Leucine-rich repeat-containing protein 59	LRRC59	11	11	3.85	3.93E-07
P40429	60S ribosomal protein L13a	RPL13A	7	1	3.79	6.94E-05
E9PKZ0	60S ribosomal protein L8	RPL8	5	5	3.77	1.49E-03
Q9Y3U8	60S ribosomal protein L36	RPL36	4	4	3.70	3.41E-04
E5RI99	60S ribosomal protein L30	RPL30	2	2	3.69	6.65E-04
P62424	60S ribosomal protein L7a	RPL7A	9	9	3.61	2.11E-03
P16403	Histone H1.2	HIST1H1C	3	3	3.54	7.88E-06
E9PF19	Transducin beta-like protein 2	TBL2	3	3	3.53	8.19E-03
O95470	Sphingosine-1-phosphate lyase 1	SGPL1	7	7	3.47	8.27E-06
P18124	60S ribosomal protein L7	RPL7	14	14	3.46	4.97E-04
E7EQV9	Ribosomal protein L15	RPL15	8	8	3.44	1.30E-03
P10412	Histone H1.4	HIST1H1E	13	4	3.38	2.39E-06
P35251-2	Replication factor C subunit 1	RFC1	7	7	3.37	1.71E-03
P47914	60S ribosomal protein L29	RPL29	3	3	3.36	2.50E-04
O00425	Insulin-like growth factor 2 mRNA-binding protein 3	IGF2BP3	11	10	3.36	3.05E-03
P35250	Replication factor C subunit 2	RFC2	5	5	3.35	6.33E-04
P46778	60S ribosomal protein L21	RPL21	6	6	3.34	9.27E-05
P62857	40S ribosomal protein S28	RPS28	4	4	3.33	9.42E-04
J3KT29	60S ribosomal protein L23	RPL23	10	10	3.32	2.09E-05
Q8IZ69-2	tRNA (uracil-5-)-methyltransferase homolog A	TRMT2A	5	5	3.27	3.25E-04

Q07020-2	60S ribosomal protein L18	RPL18	7	7	**3.26**	**2.14E-04**
J3QR09	Ribosomal protein L19	RPL19	7	7	**3.26**	**5.59E-05**
P26373	60S ribosomal protein L13	RPL13	13	13	**3.25**	**4.97E-04**
P40937-2	Replication factor C subunit 5	RFC5	5	3	**3.23**	**1.11E-03**
P16989	Y-box-binding protein 3	YBX3	7	2	**3.23**	**6.55E-05**
P05388	60S acidic ribosomal protein P0	RPLP0	11	11	**3.21**	**3.76E-03**
Q9BVJ6	U3 small nucleolar RNA-associated protein 14 homolog A	UTP14A	12	12	**3.20**	**8.45E-03**
A0A087WYC8	Regulator of nonsense transcripts 3B	UPF3B	2	2	**3.19**	**1.93E-03**
C9J4Z3	60S ribosomal protein L37a	RPL37A	2	2	**3.18**	**8.40E-04**
K7ERI7	60S ribosomal protein L22	RPL22	5	5	**3.16**	**5.82E-04**
P62851	40S ribosomal protein S25	RPS25	8	8	**3.13**	**3.15E-04**
Q96PK6	RNA-binding protein 14	RBM14	11	11	**3.13**	**8.95E-03**
P08579	U2 small nuclear ribonucleoprotein B	SNRPB2	3	3	**3.12**	**6.75E-04**
P30050	60S ribosomal protein L12	RPL12	3	3	**3.12**	**3.16E-04**
Q7Z2W4-2	Zinc finger CCCH-type antiviral protein 1	ZC3HAV1	6	6	**3.12**	**1.03E-03**
Q9H6S0	Probable ATP-dependent RNA helicase YTHDC2	YTHDC2	10	10	**3.09**	**1.24E-04**
Q9P2N5	RNA-binding protein 27	RBM27	4	4	**3.09**	**5.99E-04**
B5MCF9	Pescadillo homolog	PES1	5	5	**3.07**	**5.22E-04**
B1ANR0	Polyadenylate-binding protein 4	PABPC4	10	10	**3.06**	**6.48E-03**
Q9GZR7-2	ATP-dependent RNA helicase DDX24	DDX24	2	2	**3.05**	**9.06E-03**
P67809	Nuclease-sensitive element-binding protein 1	YBX1	13	9	**3.04**	**8.89E-04**
Q92615	La-related protein 4B	LARP4B	6	6	**3.03**	**1.07E-03**
P17844	Probable ATP-dependent RNA helicase DDX5	DDX5	22	22	**3.00**	**4.13E-03**
P62249	40S ribosomal protein S16	RPS16	16	16	**2.96**	**1.20E-03**
P62269	40S ribosomal protein S18	RPS18	15	15	**2.93**	**5.86E-04**
P46783	40S ribosomal protein S10	RPS10	14	14	**2.90**	**1.57E-05**
Q9BY77	Polymerase delta-interacting protein 3	POLDIP3	9	9	**2.90**	**3.73E-03**
P19338	Nucleolin	NCL	30	30	**2.83**	**3.62E-03**
P23396	40S ribosomal protein S3	RPS3	27	27	**2.82**	**2.38E-04**
Q96DH6	RNA-binding protein Musashi homolog 2	MSI2	3	3	**2.82**	**5.54E-03**
Q9BQG0	Myb-binding protein 1A	MYBBP1A	14	14	**2.80**	**7.79E-03**
D6RAN4	60S ribosomal protein L9	RPL9	7	7	**2.78**	**2.23E-05**
P37108	Signal recognition particle 14 kDa protein	SRP14	3	3	**2.76**	**3.48E-04**
Q14694	Ubiquitin carboxyl-terminal hydrolase 10	USP10	3	3	**2.74**	**3.52E-03**
Q9BZE4	Nucleolar GTP-binding protein 1	GTPBP4	6	6	**2.73**	**2.34E-03**
P62913	60S ribosomal protein L11	RPL11	6	2	**2.71**	**2.49E-05**
P56270-2	Myc-associated zinc finger protein	MAZ	3	3	**2.70**	**4.71E-03**
Q15233	Non-POU domain-containing octamer-binding protein	NONO	20	20	**2.70**	**7.35E-03**
P60866	40S ribosomal protein S20	RPS20	6	6	**2.67**	**3.12E-05**
Q9BQ70	Transcription factor 25	TCF25	3	3	**2.66**	**9.83E-03**
Q9P270	SLAIN motif-containing protein 2	SLAIN2	2	2	**2.65**	**2.85E-03**
Q14684-2	Ribosomal RNA processing protein 1 homolog B	RRP1B	11	11	**2.65**	**3.52E-04**
Q86XZ4	Spermatogenesis-associated serine-rich protein 2	SPATS2	5	5	**2.63**	**4.50E-04**
P62701	40S ribosomal protein S4, X isoform	RPS4X	25	25	**2.59**	**2.45E-04**
P05387	60S acidic ribosomal protein P2	RPLP2	7	7	**2.57**	**9.12E-04**
Q9NZB2-4	Constitutive coactivator of PPAR-gamma-like protein 1	FAM120A	10	10	**2.57**	**9.17E-03**
Q01081-4	Splicing factor U2AF 35 kDa subunit	U2AF1	1	1	**2.56**	**4.24E-03**
Q12906	Interleukin enhancer-binding factor 3	ILF3	11	11	**2.55**	**5.13E-03**
Q13151	Heterogeneous nuclear ribonucleoprotein A0	HNRNPA0	11	11	**2.55**	**1.39E-03**
M0R0F0	40S ribosomal protein S5	RPS5	14	14	**2.53**	**9.06E-05**
P78316	Nucleolar protein 14	NOP14	6	6	**2.53**	**1.40E-03**
Q5JR95	40S ribosomal protein S8	RPS8	9	9	**2.52**	**1.23E-04**
Q7KZI7-10	Serine/threonine-protein kinase MARK2	MARK2	4	4	**2.51**	**7.94E-03**
H0Y3N9	Histone lysine demethylase PHF8	PHF8	6	6	**2.51**	**9.71E-04**
P0CW22	40S ribosomal protein S17-like	RPS17L	10	10	**2.49**	**6.07E-05**
Q8NCA5-2	Protein FAM98A	FAM98A	4	4	**2.48**	**3.75E-04**
F8W6I7	Heterogeneous nuclear ribonucleoprotein A1	HNRNPA1	20	20	**2.48**	**7.35E-04**
P42766	60S ribosomal protein L35	RPL35	6	6	**2.47**	**3.83E-06**
Q2NL82	Pre-rRNA-processing protein TSR1 homolog	TSR1	3	3	**2.46**	**5.38E-03**
Q9BXS6-2	Nucleolar and spindle-associated protein 1	NUSAP1	8	1	**2.45**	**1.38E-03**
C9JTT7	Replication factor C subunit 4	RFC4	4	4	**2.45**	**4.97E-03**
O00411	DNA-directed RNA polymerase, mitochondrial	POLRMT	15	15	**2.43**	**3.48E-04**
P62841	40S ribosomal protein S15	RPS15	6	6	**2.41**	**2.73E-04**
F8W7C6	60S ribosomal protein L10	RPL10	6	6	**2.41**	**6.81E-04**
A0A024RAC6	Transcription elongation factor B polypeptide 3	TCEB3	9	9	**2.41**	**7.58E-04**
Q9NZI8	Insulin-like growth factor 2 mRNA-binding protein 1	IGF2BP1	19	17	**2.38**	**1.07E-03**
Q92841	Probable ATP-dependent RNA helicase DDX17	DDX17	27	22	**2.38**	**4.62E-04**
Q9HCM4-2	Band 4.1-like protein 5	EPB41L5	2	2	**2.37**	**6.23E-03**
Q96EL3	39S ribosomal protein L53, mitochondrial	MRPL53	2	2	**2.36**	**7.24E-04**
P39019	40S ribosomal protein S19	RPS19	20	20	**2.32**	**6.94E-04**
P61254	60S ribosomal protein L26	RPL26	6	6	**2.32**	**1.00E-04**
O43395	U4/U6 small nuclear ribonucleoprotein Prp3	PRPF3	6	6	**2.31**	**1.23E-03**
P46776	60S ribosomal protein L27a	RPL27A	4	4	**2.25**	**2.87E-05**
P40938-2	Replication factor C subunit 3	RFC3	4	4	**2.25**	**3.38E-03**
Q96SB4-4	SRSF protein kinase 1	SRPK1	6	6	**2.24**	**8.52E-03**
M0QZC5	40S ribosomal protein S11	RPS11	13	13	**2.24**	**3.15E-03**
Q5JTH9-2	RRP12-like protein	RRP12	7	7	**2.23**	**9.72E-04**
O94761	ATP-dependent DNA helicase Q4	RECQL4	2	2	**2.22**	**7.91E-03**
Q92522	Histone H1x	H1FX	7	7	**2.21**	**8.41E-06**
P62750	60S ribosomal protein L23a	RPL23A	11	11	**2.20**	**4.73E-06**

P25398	40S ribosomal protein S12	RPS12	5	5	**2.19**	**1.35E-04**
P11940	Polyadenylate-binding protein 1	PABPC1	21	15	**2.17**	**7.78E-03**
Q00059	Transcription factor A, mitochondrial	TFAM	9	9	**2.15**	**3.85E-03**
P62081	40S ribosomal protein S7	RPS7	11	11	**2.14**	**6.95E-04**
A0A0B4J210	La-related protein 1	LARP1	11	11	**2.12**	**2.36E-03**
C9JXB8	60S ribosomal protein L24	RPL24	4	4	**2.09**	**1.79E-04**
P62266	40S ribosomal protein S23	RPS23	3	3	**2.09**	**2.24E-03**
D3YTB1	60S ribosomal protein L32	RPL32	4	4	**2.08**	**4.48E-03**
Q13724-2	Mannosyl-oligosaccharide glucosidase	MOGS	9	9	**2.07**	**2.60E-03**
P27448-6	MAP/microtubule affinity-regulating kinase 3	MARK3	7	6	**2.04**	**3.38E-03**
P35637-2	RNA-binding protein FUS	FUS	15	13	**2.04**	**1.11E-03**
B4DLN1	39S ribosomal protein L12, mitochondrial	SLC25A10	6	6	**2.00**	**2.85E-03**
Q9BVI4	Nucleolar complex protein 4 homolog	NOC4L	5	5	**1.96**	**5.02E-03**
G3XAC6	RNA-binding protein 39	RBM39	9	9	**1.96**	**2.21E-05**
Q9NSI2-2	Protein FAM207A	FAM207A	8	8	**1.95**	**3.69E-03**
P62277	40S ribosomal protein S13	RPS13	12	12	**1.94**	**1.84E-03**
Q08211	ATP-dependent RNA helicase A	DHX9	32	32	**1.93**	**3.39E-06**
A0A087WXM6	60S ribosomal protein L17	RPL17	11	11	**1.93**	**3.06E-05**
P62753	40S ribosomal protein S6	RPS6	9	9	**1.92**	**4.63E-04**
O43172-2	U4/U6 small nuclear ribonucleoprotein Prp4	PRPF4	4	4	**1.87**	**9.76E-03**
Q14493	Histone RNA hairpin-binding protein	SLBP	2	2	**1.84**	**9.93E-03**
P51991	Heterogeneous nuclear ribonucleoprotein A3	HNRNPA3	16	3	**1.84**	**2.90E-03**
Q01844-6	RNA-binding protein EWS	EWSR1	6	6	**1.83**	**4.24E-04**
P15880	40S ribosomal protein S2	RPS2	14	14	**1.83**	**1.97E-03**
P62244	40S ribosomal protein S15a	RPS15A	9	9	**1.81**	**1.79E-03**
Q3KQU3-2	MAP7 domain-containing protein 1	MAP7D1	5	5	**1.81**	**5.38E-03**
A0A0D9SFB3	ATP-dependent RNA helicase DDX3X	DDX3X	31	29	**1.79**	**1.32E-04**
P22626	Heterogeneous nuclear ribonucleoproteins A2/B1	HNRNPA2B1	23	23	**1.78**	**5.09E-04**
H0Y6E7	RNA-binding motif protein, X chromosome	RBMX	8	8	**1.78**	**2.79E-03**
O60506-3	Heterogeneous nuclear ribonucleoprotein Q	SYNCRIP	11	8	**1.77**	**7.88E-03**
Q8WWY3	U4/U6 small nuclear ribonucleoprotein Prp31	PRPF31	6	6	**1.75**	**7.32E-03**
P63173	60S ribosomal protein L38	RPL38	7	7	**1.75**	**1.21E-03**
P62847-2	40S ribosomal protein S24	RPS24	7	7	**1.75**	**2.37E-04**
Q8IWZ3-6	Ankyrin repeat and KH domain-containing protein 1	ANKHD1	6	6	**1.74**	**9.96E-03**
O43390	Heterogeneous nuclear ribonucleoprotein R	HNRNPR	7	7	**1.73**	**1.43E-04**
Q86V81	THO complex subunit 4	ALYREF	8	1	**1.71**	**8.68E-03**
P46781	40S ribosomal protein S9	RPS9	15	15	**1.68**	**8.81E-04**
Q9H1R3	Myosin light chain kinase 2, skeletal/cardiac muscle	MYLK2	3	3	**1.66**	**9.84E-03**
P62854	40S ribosomal protein S26	RPS26	4	4	**1.65**	**4.20E-03**
Q13123	Protein Red	IK	6	6	**1.62**	**1.19E-05**
Q13895	Bystin	BYSL	9	9	**1.62**	**1.39E-03**
P62263	40S ribosomal protein S14	RPS14	9	9	**1.61**	**4.59E-03**
D6R9P3	Heterogeneous nuclear ribonucleoprotein A/B	HNRNPAB	9	9	**1.54**	**1.76E-03**
Q01105-3	Protein SET	SET	3	3	**1.51**	**5.78E-03**
Q5RKV6	Exosome complex component MTR3	EXOSC6	2	2	**1.51**	**2.40E-03**
H0YA96	Heterogeneous nuclear ribonucleoprotein D0	HNRNPD	7	5	**1.45**	**4.86E-03**
P09874	Poly [ADP-ribose] polymerase 1	PARP1	13	13	**1.37**	**1.29E-04**
Q00839-2	Heterogeneous nuclear ribonucleoprotein U	HNRNPU	28	28	**1.37**	**5.02E-03**
A0A087X2D0	Serine/arginine-rich splicing factor 3	SRSF3	5	5	**1.13**	**1.33E-04**
B4DY09	Interleukin enhancer-binding factor 2	ILF2	6	6	**1.02**	**1.71E-03**

Supplementary Table S2

Binding free energy differences ($\Delta\Delta \boldsymbol{G}$ in kcal/mol) of TARG1 mutants in complex with ADPr ($\Delta\Delta \boldsymbol{G} = \Delta \boldsymbol{G}_{\text{wildtype}} - \Delta \boldsymbol{G}_{\text{mutant}}$).

	**E** [b]	**L**	**R**
**G19** [a]	12.8	13.2	13.3
**D20**	12.9	10.1	8.7
**L21**	-10.2	/	25.6
**F22**	8.8	0.5	1.2
**H32**	12.7	6.0	17.8
**C33**	2.8	4.7	4.3
**I34**	0.1	4.6	5.5
**S35**	-12.5	-6.5	5.4
**R39**	11.1	-9.3	/
**M40**	11.8	3.1	13.9
**A42**	0.0	-9.7	4.5
**G43**	**-16.3** [c]	**-15.1**	**-15.7**
**I44**	**-18.9**	-10.7	**-18.9**
**A45**	**-18.9**	11.5	**-18.9**
**L47**	10.1	/	5.1
**T83**	6.6	4.7	-6.6
**A87**	-8.3	-9.6	6.1
**R119**	8.6	11.8	/
**I120**	3.4	6.6	13.8
**G121**	2.5	15.7	18.8
**C122**	10.7	3.6	-9.3
**G123**	**-18.9**	**-18.9**	**-18.9**
**L124**	5.1	/	6.1
**D125**	-8.4	12.6	12.1
**Y150**	10.9	12.9	12.2
**T151**	10.6	11.6	12.7
**L152**	6.0	/	11.4

[a] The residues of TARG1 within ADPr binding pocket (by 5 Å).
[b] Each residue was mutated to glutamate (E), leucine (L), and arginine (R).
[c] The potent mutations ($\Delta\Delta \boldsymbol{G}$ < -15 kcal/mol) were highlighted in bold fonts.

Supplementary Table S3

Complete gene ontology analyses of TARG1 interacting proteins

Proteins that were upregulated at least 10-fold in TAP-TARG1 pulldowns over TAP-tag alone with and without olaparib were analyzed for statistical overrepresentation of gene ontology (GO) terms using PANTHER GO-Slim annotation data sets (PANTHER version 13.0, release 2017-11-12, analysis type PANTHER Overrepresentation Test, released 20171205) for "biological process", "molecular function" or "cellular component", sorted by false discovery rate (FDR; Reference list: Homo sapiens, test type: Fisher's Exact with FDR multiple test correction, FDR<0.05).

+ Olaparib	Homo sapiens REF #	Input #	Expected	Fold enrichment	Raw p-value	FDR
PANTHER GO-Slim Biological Process						
translation (GO:0006412)	141	10	.42	24.07	2.04E-11	4.97E-09
cellular component biogenesis (GO:0044085)	504	13	1.49	8.75	2.74E-09	3.34E-07
rRNA metabolic process (GO:0016072)	111	6	.33	18.35	1.22E-06	7.43E-05
cellular component organization or biogenesis (GO:0071840)	1685	18	4.96	3.63	1.14E-06	9.27E-05
RNA metabolic process (GO:0016070)	1023	11	3.01	3.65	1.89E-04	9.23E-03
biosynthetic process (GO:0009058)	1659	14	4.89	2.86	2.85E-04	1.16E-02
organelle organization (GO:0006996)	1152	11	3.39	3.24	5.17E-04	1.80E-02
nucleobase-containing compound metabolic process (GO:0006139)	2050	15	6.04	2.48	7.52E-04	2.29E-02
chromatin organization (GO:0006325)	255	5	.75	6.65	9.97E-04	2.70E-02
primary metabolic process (GO:0044238)	4346	24	12.81	1.87	1.34E-03	3.27E-02
PANTHER GO-Slim Molecular Function						
structural constituent of ribosome (GO:0003735)	123	18	.36	49.67	3.31E-25	6.28E-23
structural molecule activity (GO:0005198)	480	18	1.41	12.73	2.69E-15	2.56E-13
RNA binding (GO:0003723)	415	11	1.22	9.00	4.02E-08	2.54E-06
nucleic acid binding (GO:0003676)	1612	17	4.75	3.58	2.93E-06	1.39E-04
DNA binding (GO:0003677)	262	6	.77	7.77	1.34E-04	5.11E-03
RNA helicase activity (GO:0003724)	34	3	.10	29.95	1.75E-04	5.53E-03
mRNA binding (GO:0003729)	117	4	.34	11.60	4.44E-04	1.05E-02
helicase activity (GO:0004386)	67	3	.20	15.20	1.15E-03	2.43E-02
Unclassified (UNCLASSIFIED)	12190	22	35.92	.61	4.37E-04	1.18E-02
PANTHER GO-Slim Cellular Component						
ribosome (GO:0005840)	156	23	.46	50.04	2.37E-32	7.46E-31
ribonucleoprotein complex (GO:0030529)	415	29	1.22	23.72	2.03E-32	1.28E-30
cytosol (GO:0005829)	468	24	1.38	17.40	1.63E-23	3.42E-22
macromolecular complex (GO:0032991)	2153	32	6.34	5.04	4.90E-16	7.71E-15
organelle (GO:0043226)	3734	33	11.00	3.00	3.13E-10	3.94E-09
cytoplasm (GO:0005737)	2927	28	8.62	3.25	2.95E-09	3.09E-08
intracellular (GO:0005622)	4976	36	14.66	2.46	7.30E-09	6.57E-08
cell part (GO:0044464)	6050	36	17.83	2.02	1.69E-06	1.33E-05
Unclassified (UNCLASSIFIED)	14246	25	41.98	.60	1.44E-05	1.01E-04
nucleolus (GO:0005730)	113	5	.33	15.02	2.52E-05	1.59E-04
- Olaparib						
PANTHER GO-Slim Biological Process						
DNA metabolic process (GO:0006259)	381	12	.65	18.41	1.25E-12	3.05E-10
DNA repair (GO:0006281)	162	8	.28	28.86	3.79E-10	4.62E-08
nitrogen compound metabolic process (GO:0006807)	2435	18	4.17	4.32	1.64E-08	1.33E-06
response to stress (GO:0006950)	684	10	1.17	8.55	1.64E-07	1.00E-05
nucleobase-containing compound metabolic process (GO:0006139)	2050	15	3.51	4.28	5.28E-07	2.58E-05
chromatin organization (GO:0006325)	255	6	.44	13.75	4.85E-06	1.97E-04
response to abiotic stimulus (GO:0009628)	75	4	.13	31.17	9.83E-06	3.43E-04

organelle organization (GO:0006996)	1152	9	1.97	4.57	1.10E-04	3.36E-03
metabolic process (GO:0008152)	5416	20	9.27	2.16	1.60E-04	3.91E-03
DNA replication (GO:0006260)	156	4	.27	14.99	1.56E-04	4.23E-03
biosynthetic process (GO:0009058)	1659	10	2.84	3.52	3.54E-04	7.85E-03
cellular component organization (GO:0016043)	1543	9	2.64	3.41	9.47E-04	1.93E-02
primary metabolic process (GO:0044238)	4346	16	7.44	2.15	1.36E-03	2.55E-02
cellular component organization or biogenesis (GO:0071840)	1685	9	2.88	3.12	1.76E-03	3.06E-02
chromatin assembly (GO:0031497)	40	2	.07	29.23	2.34E-03	3.81E-02
regulation of cell cycle (GO:0051726)	164	3	.28	10.69	2.88E-03	4.39E-02
DNA recombination (GO:0006310)	49	2	.08	23.86	3.43E-03	4.92E-02
PANTHER GO-Slim Molecular Function						
DNA binding (GO:0003677)	262	10	.45	22.31	2.05E-11	3.90E-09
nucleic acid binding (GO:0003676)	1612	12	2.76	4.35	9.18E-06	8.72E-04
PANTHER GO-Slim Cellular Component						
chromosome (GO:0005694)	201	11	.34	31.99	3.96E-14	2.49E-12
nucleus (GO:0005634)	1839	18	3.15	5.72	1.82E-10	5.75E-09
nuclear chromosome (GO:0000228)	136	7	.23	30.08	4.04E-09	8.49E-08
organelle (GO:0043226)	3734	18	6.39	2.82	1.05E-05	1.65E-04
protein-DNA complex (GO:0032993)	31	3	.05	56.56	2.64E-05	3.33E-04
intracellular (GO:0005622)	4976	18	8.51	2.11	5.92E-04	6.21E-03
macromolecular complex (GO:0032991)	2153	10	3.68	2.71	2.64E-03	2.38E-02

Supplementary Methods

<u>*In silico* mutational analyses</u>

Conservation Analyses. Multiple sequence alignment on the residues of the ADPr binding sites were carried out among TARG1, MacroD2, MacroD1, AF1521 and MacroH2A using Clustal Omega [4]. From this procedure, three conserved or strongly similar residues across the selected macrodomains were identified, G43, I44 and G123 (Supplementary Fig S1 B).

In silico alanine scanning. The ABS-Scan web-server [5] systematically evaluates amino acids for their importance in protein-ligand interactions by in silico alanine-scanning mutagenesis. The crystal structure of TARG1 (PDBID 4J5S) was used as starting structure [6]. A distance cut-off of 5 Å was chosen to define the binding site around the ADPr. For each residue within the cut-off, all side chain atoms beyond C_β were removed and the missing hydrogen was added, obtaining an alanine side chain. The method relies on two assumptions: (i) The introduced point mutation does not drastically change the structure of the protein and (ii) the mode of ligand interaction is unchanged [5]. Modeler library was used on all selected residues, coupled with steps of energy minimization to ensure that no steric clashes occur between protein and ligand atoms [7]. The structural quality of the generated protein structures was estimated through Discrete Optimized Protein Energy (DOPE) score [8], while the energetics of a protein-ligand complex was scored by using Autodock 4.1 forcefield [9]. The contribution of a specific amino acid is determined by the difference in interaction score of mutant and wild-type protein (ΔΔG value). From this procedure, I44 emerged as relevant residues for ADPr binding (Supplementary Fig S1 C).

Computational mutagenesis. The residues of TARG1 ADPr binding pocket were systematically mutated to glutamate (E), leucine (L), and arginine (R) by using the Swiss-PdbViewer package [10]. In this procedure, the input coordinates of the different amino acids for each residue are minimized using the conjugate gradient method to remove poor contacts. This is followed by molecular dynamics simulation (Langevin dynamics at constant temperature), and a short minimization to obtain the final energy of the system. The AMBER score [11] is calculated as E(Complex) - [E(protein) + E(Ligand)]. The entropic contribution is supposed to be constant since it is assumed that point mutations in the protein do not significantly affect the conformation of the mutated protein, as discussed previously [12]. AMBER score implements molecular mechanics Generalized Born/surface area simulations with traditional general AMBER force field for ligand molecules [13]. The interaction between

the ligand and the protein is represented by electrostatic and van der Waals energy terms, and the solvation energy is calculated using Generalized Born solvation model. From this procedure, the most powerful mutants were the ones from G43, I44, A45, G123 (Supplementary Table S2).

Hydrolase assay

Hydrolase assays were performed as described previously [14].

WST-1 cell proliferation assay

$3x10^3$ cells were seeded per well of a 96-well plate -/+ 500 ng/ml doxycycline in triplicates. Cell proliferation was measured every 24 h over a course of 4 days using the cell proliferation reagent WST-1 (Roche) according to manufacturer's instructions.

Colony formation assay

Stable HeLa Flp-In™ T-REx™ cells, which were or were not induced with 500 ng/ml doxycycline for 72 h to induce transgene or shRNA expression, were seeded at a density of $1x10^5$ cells per well in 6-well plates. After 24 h, the cells were treated with hydroxyurea, etoposide or doxorubicin at the indicated concentrations or with diluent as a control. The cell culture medium was exchanged after 24 h (doxorubicin) or after 48 h (hydroxyurea, etoposide) with fresh medium (-/+ doxycycline). The cells were fixed and stained on day 4 after seeding with methylene blue (0.2% in methanol). Cell density was measured using Adobe Photoshop (CS5 Extended V12.0, Adobe Systems Incorporated) and normalized to the cell density of untreated or DMSO-treated cells as a measure for relative cell survival.

SUnSET assay [3]

Cells were seeded at a density of $1x10^5$ cells per well of a 6-well plate and were or were not treated with 100 ng/ml doxycycline to induce HA-TARG1 expression for 48 h. The cells were treated with 10 µg/ml puromycin for 10 min at 37°C and were then washed twice with PBS before fresh cell culture medium was added. The cells were incubated for additional 50 min at 37°C, washed with PBS and were harvested for the preparation of whole cell lysates. As a control, cells were treated with 25 µg/ml cycloheximide throughout the 10 min pulse labeling period and the 50 min chase period to inhibit translation. An equal volume of each cell lysate was analyzed by SDS-PAGE and Western blotting for incorporation of puromycin into nascent polypeptides using an anti-puromycin antibody (12D10, 1:20,000).

References

1 Peterson, F. C. *et al.* Orphan macrodomain protein (human C6orf130) is an O-acyl-ADP-ribose deacylase: solution structure and catalytic properties. *J Biol Chem* **286**, 35955-35965, doi:10.1074/jbc.M111.276238 (2011).

2 Niesen, F. H., Berglund, H. & Vedadi, M. The use of differential scanning fluorimetry to detect ligand interactions that promote protein stability. *Nature protocols* **2**, 2212-2221, doi:10.1038/nprot.2007.321 (2007).

3 Schmidt, E. K., Clavarino, G., Ceppi, M. & Pierre, P. SUnSET, a nonradioactive method to monitor protein synthesis. *Nature methods* **6**, 275-277, doi:10.1038/nmeth.1314 (2009).

4 Sievers, F. *et al.* Fast, scalable generation of high-quality protein multiple sequence alignments using Clustal Omega. *Molecular systems biology* **7**, 539, doi:10.1038/msb.2011.75 (2011).

5 Anand, P., Nagarajan, D., Mukherjee, S. & Chandra, N. ABS-Scan: In silico alanine scanning mutagenesis for binding site residues in protein-ligand complex. *F1000Research* **3**, 214, doi:10.12688/f1000research.5165.2 (2014).

6 Sharifi, R. *et al.* Deficiency of terminal ADP-ribose protein glycohydrolase TARG1/C6orf130 in neurodegenerative disease. *Embo J* **32**, 1225-1237, doi:10.1038/emboj.2013.51 (2013).

7 Sali, A. & Blundell, T. L. Comparative protein modelling by satisfaction of spatial restraints. *J Mol Biol* **234**, 779-815, doi:10.1006/jmbi.1993.1626 (1993).

8 Shen, M. Y. & Sali, A. Statistical potential for assessment and prediction of protein structures. *Protein Sci* **15**, 2507-2524, doi:10.1110/ps.062416606 (2006).

9 Huey, R., Morris, G. M., Olson, A. J. & Goodsell, D. S. A semiempirical free energy force field with charge-based desolvation. *Journal of computational chemistry* **28**, 1145-1152, doi:10.1002/jcc.20634 (2007).

10 Schwede, T., Kopp, J., Guex, N. & Peitsch, M. C. SWISS-MODEL: An automated protein homology-modeling server. *Nucleic Acids Res* **31**, 3381-3385 (2003).

11 Graves, A. P. *et al.* Rescoring docking hit lists for model cavity sites: predictions and experimental testing. *J Mol Biol* **377**, 914-934, doi:10.1016/j.jmb.2008.01.049 (2008).

12 Massova, I. & Kollman, P. A. Computational Alanine Scanning To Probe Protein−Protein Interactions: A Novel Approach To Evaluate Binding Free Energies. . *Am. Chem. Soc.* **121**, 8133-8143 (1999).

13 Wang, J., Wolf, R. M., Caldwell, J. W., Kollman, P. A. & Case, D. A. Development and testing of a general amber force field. *Journal of computational chemistry* **25**, 1157-1174, doi:10.1002/jcc.20035 (2004).

14 Eckei, L. *et al.* The conserved macrodomains of the non-structural proteins of Chikungunya virus and other pathogenic positive strand RNA viruses function as mono-ADP-ribosylhydrolases. *Sci Rep* **7**, 41746, doi:10.1038/srep41746 (2017).
